# Whole-Genome Comparisons Among the Genus *Shewanella* Reveal the Enrichment of Genes Encoding Ankyrin-Repeats Containing Proteins in Sponge-Associated Bacteria

**DOI:** 10.3389/fmicb.2019.00005

**Published:** 2019-02-06

**Authors:** Anoop Alex, Agostinho Antunes

**Affiliations:** ^1^CIIMAR/CIMAR, Interdisciplinary Centre of Marine and Environmental Research, University of Porto, Porto, Portugal; ^2^Department of Biology, Faculty of Sciences, University of Porto, Porto, Portugal

**Keywords:** sponge-microbe symbiosis, *Shewanella*, adaptation, eukaryotic-like proteins, ankyrin repeat

## Abstract

The bacterial members of the genus *Shewanella* are widely distributed and inhabit both freshwater and marine environments. Some members of *Shewanella* have gained considerable attention due to its ability to survive in redox-stratified environments. However, a gap of knowledge exists on the key genomic features of the sponge-associated *Shewanella* sp. involving the successful host-bacteria interaction, as sponge-symbiotic *Shewanella* are largely underrepresented in the public repositories. With the aim of identifying the genomic signatures of sponge-*Shewanella* association, we generated a high-quality genome data of a sponge-associated, *Shewanella* sp. OPT22, isolated from the intertidal marine sponge *Ophlitaspongia papilla* and performed comprehensive comparative analyses of 68 genome strains of the genus *Shewanella* including two previously reported genomes of sponge-associated bacteria, *Shewanella spongiae* KCTC 22492 and *Shewanella* sp. Alg231_23. The 16S rRNA-based phylogenetic reconstruction showed the well-supported affiliation of OPT22 and KCTC 22492 with previously reported sponge-associated bacteria, affirming the “sponge-specific” nature of these two bacterial strains isolated from different marine sponge species from the Atlantic and Pacific (East Sea) Oceans, respectively. The genome comparison of the 68 strains of *Shewanella* inhabiting different habitats revealed the unusual/previously unreported abundance of genes encoding for ankyrin-repeat containing proteins (ANKs) in the genomes of the two sponge-associated strains, OPT22 (ANKs; *n* = 45) and KCTC 22492 (ANKs; *n* = 52), which might be involved in sponge-*Shewanella* interactions. Focused analyses detected the syntenic organization of the gene cluster encoding major secretion system (type III/IV/VI) components and the presence of effector homologs in OPT22 and KCTC 22492 that seem to play a role in the virulence of the sponge bacteria. The genomic island (GI) of *Shewanella* sp. OPT22 was identified to localize a gene cluster encoding T4SS components and ANK (*n* = 1), whereas *S. spongiae* KCTC 22492 harbored a total of seven ANKs within multiple GIs. GIs may play a pivotal role in the dissemination of symbioses-related genes (ANKs) through the horizontal gene transfer, contributing to the diversification and adaptation of sponge-associated *Shewanella*. Overall, the genome analyses of *Shewanella* isolates from marine sponges revealed genomic repertoires that might be involved in establishing successful symbiotic relationships with the sponge hosts.

## Introduction

Members of the genus *Shewanella* (class *Gammaproteobacteria*) are facultative anaerobic bacteria widely detected in both marine and freshwater ecosystems. Ability to utilize various electron acceptors in the absence of oxygen is the hallmark feature of the genus *Shewanella*. It enabled them to adapt and thrive successfully in diverse habitats, ranging from varied salt concentrations, barometric pressures, deep-cold marine environments, and hydrothermal vents. *Shewanella* genus gained much attention from the scientific community, and has been of great interest due to the ability of several members of the genus *Shewanella* to convert heavy metals and toxic substances (e.g., uranium, chromium, mercury, arsenic, technetium, and cobalt) to least harmful products, thus making them suitable candidates for bioremediation (Hau and Gralnick, 2007). Moreover, they play a significant role in global biogeochemical cycles of C, N, and S in redox-stratified marine environments (Brettar et al., 2001).

*Shewanella* species that exist in different ecosystems are reported to have several lifestyles, mainly, the most common free-living state, pathogens (infectious agents in humans, fishes), and epibionts (living on the surface of other organisms), such as *S*. *marinintestina*, *S*. *schlegeliana*, and *S*. *sairae* isolated from the squid intestine and several fishes. The ecological roles of *Shewanella* living in different habitats are described in detail elsewhere (Hau and Gralnick, 2007; Janda and Abbott, 2014). Symbiotic associations among the members of the genus *Shewanella* were reported between *S*. *pealeana* and accessory nidamental glands (ANG) of the cephalopod, *Loligopealei* (squid). This symbiotic association may provide protection to eggs from predation or infection through the production of protective compounds (Barbieri et al., 2001). In marine environments, *Shewanella* are known to colonize other invertebrate hosts like the sea cucumber *Apostichopus japonicus* (Enomoto et al., 2012; Hong et al., 2017), the marine hydroid *Hydractinia echinata* (Rischer et al., 2016), the sipuncula *Phascolosoma japonicum* (“peanut worm”) and hydrocoral species (Ivanova et al., 2004), the red sea coral of the genus *Favia* (Shnit-Orland et al., 2010), and sponges (Lee et al., 2006; Yang et al., 2006).

Sponges (phylum *Porifera*) are known to harbor abundant and diverse communities of symbiotic microorganisms. Several approaches including the whole-genome sequencing of symbiotic microbes isolated from sponges, metagenomics/metatranscriptomics, and single-cell genomics have provided insight into the genomic mechanisms of sponge-microbe association. For instance, the abundance and role of transposable insertion elements in the evolution of symbiotic bacterial genomes (Thomas et al., 2010); frequent detection of the genes encoding for adhesion-related proteins and eukaryotic-like proteins (ELPs) and the role of secretion systems (SSs) and effector molecules (Fan et al., 2012; Liu et al., 2012; Bondarev et al., 2013; Alex and Antunes, 2015; Romano et al., 2016) were described as key features.

Despite these advances in the understanding of symbiotic bacteria associated with several sponge species, no comparative genomic evidence is yet available for the most widely studied members of the genus *Shewanella*, particularly those living in close association with sponge hosts. So far, the genomes of only two strains- *Shewanella* sp. Alg231_23 (PRJEB13410) isolated from an Atlantic sponge *Spongia officinalis* and *Shewanella spongiae* KCTC 22492 isolated from a marine sponge living at 20 m water depth of the East Sea, Korea (Yang et al., 2006) are available.

Here, we performed comprehensive comparative analyses of 68 genome strains of the genus *Shewanella* including three sponge-associated bacteria, the previously reported *S*. *spongiae* KCTC 22492 and *Shewanella* sp. Alg231_23, and we further generated a high-quality genome data of a sponge-associated bacterium, *Shewanella* sp. OPT22, to investigate the genomic features responsible for establishing a successful symbiotic sponge-bacteria relationship. Our study showed: (i) the sponge-specific clustering of two sponge-associated *Shewanella* (OPT22 and KCTC 22492), (ii) an abundance of the genes encoding for ankyrin-repeat containing proteins (ANKs) responsible for evading the host immune response in the genome of OPT22 (ANKs; *n* = 45) and KCTC 22492 (ANKs; *n* = 52), (iii) localization of ANKs within the genomic islands (GIs) suggesting the role of horizontal gene transfer to enhance the ecological fitness of the symbiotic bacteria, and (iv) several SS components/effector proteins that act as virulence factors to modulate the bacteria-host interactions. To our knowledge, this is the first detailed genomic analyses of *Shewanella* spp. isolated from marine sponges unraveling the unique genomic repertoire for its successful colonization and symbiotic lifestyle.

## Materials and Methods

### Isolation of *Shewanella*

*Shewanella* sp. OPT22 was isolated from the intertidal marine sponge *Ophlitaspongia papilla* (class *Demospongiae*) collected from the Atlantic coast (41.2308206N 8.7216926W) of Portugal. Sponges were collected in Ziploc^®^bags and transported to the lab in natural seawater (in a cooling box) within 1 h, and processed immediately. In the lab, ≈1 cm^3^ of the sponge tissue was carefully excised using a sterile surgical blade and processed under dissection microscope to remove any external debris or other macroscopic materials. Furthermore, sponge tissue was washed using natural sterile seawater (NSW) for 3–4 times on a horizontal platform shaker for 5 min at room temperature in order to remove the loosely associated microbes and other debris. Tissue sample was grinded and homogenized using a sterile pestle and mortar with 10 ml NSW. The homogenate was serially diluted (100 μl of the 10^-1^ through 10^-5^ dilutions) and spread plated on Difco^TM^ Marine Agar 2216 medium containing amphotericin B (2.5 mg/L). Amphotericin B was used to prevent the fugal growth. All plates were incubated in dark at 28°C for 3–4 days. Single colonies were obtained after repeated streaking. Bacterial colonies were inoculated into 5 ml tubes containing Difco^TM^ Marine Broth 2216 and kept under constant shaking at 28°C. Genomic DNA was extracted from the bacterial cultures in stationary phase using PureLink^TM^ Genomic DNA kit (Invitrogen) according to the manufacturer’s protocol for bacterial DNA isolation.

### Genome Sequencing, Assembly, and Annotation

Paired-end (PE) read library (2 x 100nt) of *Shewanella* sp. OPT22 genomic DNA was constructed with an insert size of ∼350 bp and sequencing was performed using the Illumina’s HiSeq 2500 Sequencing System. Low quality reads (Phred score < 30) and adapter sequences were removed using cutadapt v1.12 (Martin, 2011). The processed reads of > 1700-fold coverage (based on a 5 Mb genome size) was assembled using Velvet v1.2.10 (Zerbino and Birney, 2008) with the best possible *k-mer* coverage value (*k* = 99) obtained from VelvetK. Gene prediction was performed using PROKKA v1.12 (Seemann, 2014). Hidden Markov Model (HMM) profiles of Pfam31.0 and TIGRFAMs15.0, and curated dataset constituting bacterial protein sequences from the UniProt Knowledgebase (UniProtKB Release 2017_3) were compiled locally for the annotation of predicted coding sequences (CDS). Genome completeness was estimated using *Alteromonadales* marker gene sets defined by CheckM (Parks et al., 2015).

Proteins were categorized in clusters of orthologous groups (COGs) with standalone RPS-BLAST v2.2.31 (Reverse Position-Specific BLAST) against NCBI preformatted CDD (conserved domains database^1^) with an *E*-value cutoff of 1e-03. The output was parsed-out and classified into different categories using *cdd2cog.pl* script v0.1 (Andreas, 2016). Significant differences in proportion of COG categories were determined using *z*-test. A total of 67 bacterial genomes of the genus *Shewanella* were retrieved using NCBI Genome Downloading Scripts^2^. The genome of *S. spongiae* KCTC 22492 was accessed on October 2018. The list of the genomes of the 67 *Shewanella* spp. isolated from different ecological niches and assembly version of the genomes used for our comparative analyses is given in Supplementary Table S1. The genomes were re-annotated using PROKKA v1.12 (Seemann, 2014) in order to avoid the inconsistencies with different annotation pipelines.

### Phylogenetic Analyses of the Genus *Shewanella*

The 16S ribosomal RNA (16S rRNA) gene sequences were retrieved from the genomes of *Shewanella* considered in the present study (the 16S rRNA genes were identified only in the genomes of 57 *Shewanella* spp.) along with the 16S rRNA sequences of the sponge-associated *Shewanella* (retrieved by BLAST; *n* = 11) and were aligned with Clustal Omega (Sievers and Higgins, 2014) implemented in SeaView v4.4.2 (Gouy et al., 2010), with ambiguous regions removed by Gblocks v0.91b (Talavera and Castresana, 2007) using “less stringent” options. The final alignment (∼1,458 bps) was used to construct a maximum likelihood phylogenetic tree in PhyML (Guindon et al., 2010); with 500 bootstrap replicates using nearest-neighbor interchanges (NNIs) tree search criteria. Evolutionary model GTR + I + G adopted under Akaike Information Criterion with correction (AICc) implemented in MrAIC v1.4.4 (Nylander, 2004) was used as a best-fit model of nucleotide substitution. The Pyani (Pritchard et al., 2016) package was used to measure the average nucleotide identity (ANI) and the genome-scale relatedness of bacterial members.

The whole-genome phylogenetic tree was constructed using protein sequences of 114 conserved core single-copy orthologous genes (encompassing 28,356 amino acids). Briefly, the “core” genes of the members of the genus *Shewanella* was estimated by clustering the CDS using the bidirectional best-hit (BDBH), COGtriangles, and OrthoMCL clustering algorithms implemented in GET_HOMOLOGUES v18042017 (Contreras-Moreira and Vinuesa, 2013) with 75% pairwise alignment coverage and *E*-value (expectation value for DIAMOND BLASTP alignments) set at 1e-03. Inparalogs were excluded from the clusters (option “-e”). A total of 114 single-copy orthologous genes were aligned using MUSCLE v3.8.31 (Edgar, 2004) and low quality alignment regions were removed using trimAl v1.4 with “automated1” option (Capella-Gutiérrez et al., 2009). Alignment files were concatenated to a super-alignment using FASconCAT v1.1 (Kück and Meusemann, 2010). A phylogenetic tree was inferred using maximum likelihood method implemented in IQ-TREE v1.6.1 (Nguyen et al., 2015) under automated model selection option “TEST” with 1000 bootstrap replicates statistical support.

### Detection of Eukaryotic-Like Proteins (ELPs)

Eukaryotic-like proteins (ELPs) containing motifs- tetratrico peptide repeats (TPRs: IPR011990, IPR019734, IPR013105, IPR001440, and IPR011717), ankyrin repeats (ANKs: IPR020683 and IPR002110), and Sel1 repeats (IPR006597)-were detected using InterProScan v5.24.63 (Jones et al., 2014). Presence of TPRs and Sel1 was further validated using TPRpred with *E*-value cutoffs of 1e-03 and 1e-02. Significant differences in proportion of ANKs were determined using *z*-test.

Genomic islands were predicted using an integrated approach implemented in IsandViewer 4 (Bertelli et al., 2017). Pre-computed complete reference genome of *Shewanella oneidensis* MR-1 chromosome available in the IslandViewer webserver was selected to re-order the contigs. Subcellular localization of the genes in the GI was performed using PSORTb v3.0.2 (Yu et al., 2010).

### Identification of Secretion System Components

Secretion system components were identified by uploading the whole proteome of *Shewanell*a sp. OPT22 as a query and performed BLAST search using BlastKOALA tool implemented in KEGG v2.1 (Kanehisa et al., 2016, 2017) against the taxonomic group “Bacteria” and “genus_prokaryote” database. T346Hunter, a web based tool was also used to predict the encoded SSs (Martínez-García et al., 2015). The predicted genes were further confirmed by performing NCBI BLASTP service. Easyfig v2.2.2 (Sullivan et al., 2011) was used to generate the gene clusters of the SSs and the GI.

## Results and Discussion

### Phylogeny of the Genus *Shewanella* and the Evidence for Sponge-Specific Clustering of Two Symbiotic Strains

**FIGURE 1:**
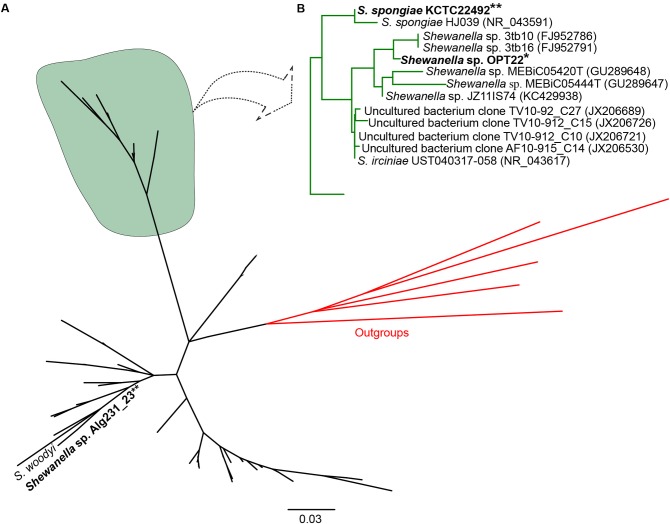
Phylogeny of the genus *Shewanella*. **(A)** Phylogenetic tree inferred using the 16S rRNA gene sequences of *Shewanella* species used for the comparative genomics and the closely related species retrieved from best BLAST hit (shown in Table 1). The sponge-specific cluster is shaded in green. Outgroup species are labeled in red. **(B)** Detailed view of the sponge-specific cluster. Strains in bold denote the only available genomes of sponge-associated *Shewanella* that are used in this study. The *Shewanella* sp. OPT22 sequenced in this study is highlighted by a single asterisk, grouped with other sponge-associated *Shewanella* strains. *Shewanella* sp. 3tb10 and *Shewanella* sp. 3tb16 were isolated from a coral host. Strains with two asterisks denote previously reported sponge-associated *Shewanella*. The complete phylogenetic tree is provided in Supplementary Figure S1.

Initial similarity search using BLASTn revealed a higher relatedness (sequence identity ranging from 97 to 99%) of the 16S rRNA gene sequences of *Shewanella* sp. OPT22 sequenced in this study with uncultured bacteria isolated from the sponge *Ircinia* spp. (Northwestern Mediterranean Sea; JX206530, JX206726, JX206689, and JX206721), *Shewanella* strains isolated from the sponge *Ircinia dendroides* (Mediterranean Sea; NR_043617), the Micronesian sponge (GU289647–GU289648), *I*. *strobilina* (KC429938), and the Sea of Japan (NR_043591); and *Shewanella* strains isolated from the coral *Montastrea annularis* collected in the Florida keys (Table 1). Phylogenetic analysis using the 16S rRNA gene sequences revealed the clustering of *Shewanella* sp. OPT22 isolated from the Atlantic sponge *O. papilla* and *S. spongiae* KCTC 22492 isolated from a marine sponge collected in Korea with other geographically separated sponge-and coral-associated bacteria, which favor the sponge-specific nature of *Shewanella* sp. OPT22 and *S*. *spongiae* KCTC 22492, and likely to represent true sponge-associated bacteria (Figure 1 and Supplementary Figure S1). Clustering of 16S rRNA gene sequences recovered from geographically isolated sponges and/or from different sponge species was reported in many studies (Simister et al., 2012; Alex et al., 2013; Thomas et al., 2016).

**Table 1:** Best BLAST hit identities of the queried 16S rRNA gene of *Shewanella* sp. OPT22.

Accession	Description	Query coverage	*E*-value	Identity	Isolation source
JX206726	Uncultured bacterium clone TV10-912_C15	97%	0	99%	*Ircinia* spp.
JX206721	Uncultured bacterium clone TV10-912_C10	97%	0	99%	*Ircinia* spp.
JX206530	Uncultured bacterium clone AF10-915_C14	97%	0	99%	*Ircinia* spp.
JX206689	Uncultured bacterium clone TV10-92_C27	97%	0	99%	*Ircinia* spp.
NR_043617	*Shewanella irciniae* strain UST040317-058	96%	0	99%	*I. dendroides*
GU289648	*Shewanella* sp. MEBiC05420T	99%	0	98%	Marine sponge
GU289647	*Shewanella* sp. MEBiC05444T	98%	0	98%	Marine sponge
NR_043591	*Shewanella spongiae* strain HJ039	98%	0	97%	Marine sponge
FJ952791	*Shewanella* sp. 3tb16	92%	0	99%	*Montastrea annularis*
FJ952786	*Shewanella* sp. 3tb10	91%	0	99%	*M. annularis*
KC429938	*Shewanella* sp. JZ11IS74	90%	0	99%	*I. strobilina*


**FIGURE 2:**
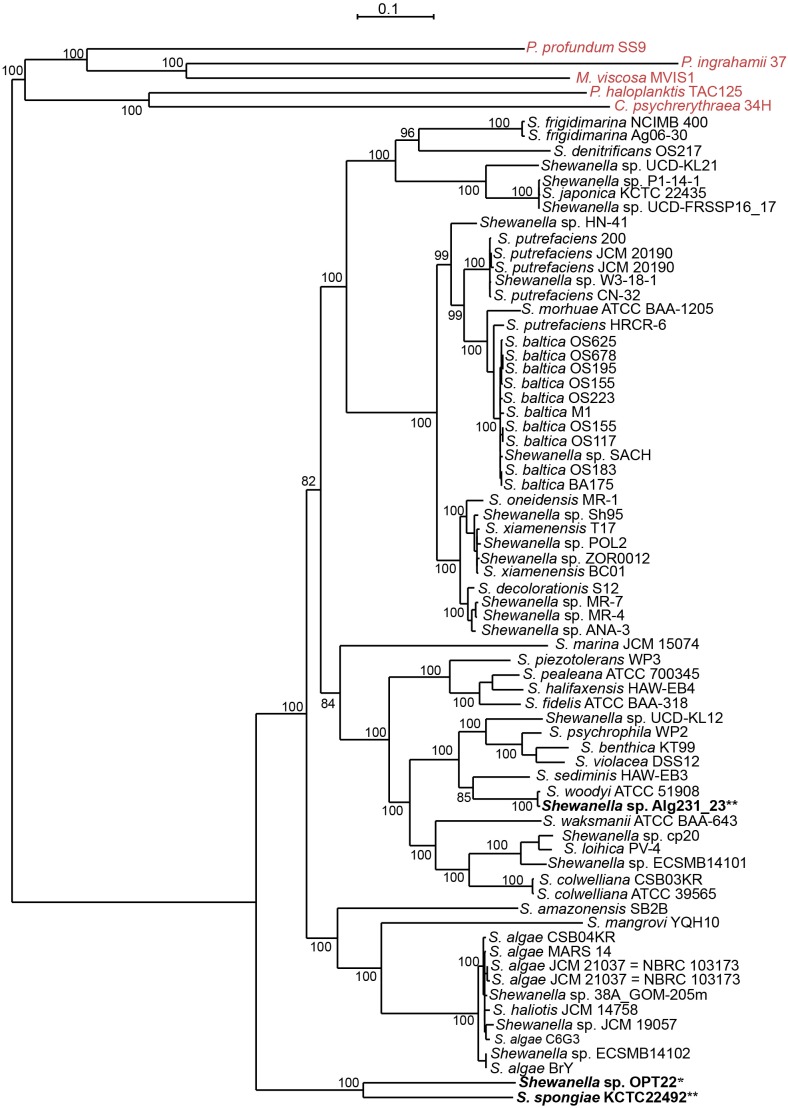
Whole-genome phylogeny of the genus *Shewanella*. A maximum likelihood tree was inferred from a concatenated protein dataset of 114 single-copy orthologous genes. Strains in bold denote the only available genomes of sponge-associated *Shewanella* that are used in this study. The genomes of the *Shewanella* strains sequenced here and from previous studies are shown as single and double asterisks, respectively. Outgroup species are labeled in red. Bootstrap node support values above 80 are shown.

However, *Shewanella* sp. Alg231_23 isolated from the sponge *Spongia officinalis* sampled from the Atlantic coast of Portugal grouped with *S*. *woodyi* ATCC 51908 isolated from a squid/seawater from depths of 200–300 m in the Alboran Sea (Figure 1 and Supplementary Figure S1). The whole-genome phylogeny using a concatenated set of 114 single-copy orthologous genes also grouped *Shewanella* sp. Alg231_23 and *S*. *woodyi* ATCC 51908 together, whereas *Shewanella* sp. OPT22 and *S*. *spongiae* KCTC 22492 were clustered together (Figure 2). Supporting the observed phylogenetic pattern, the ANI analysis also revealed the close evolutionary relatedness of *Shewanella* sp. Alg231_23 and *S*. *woodyi* ATCC 51908, with their ANI values being ∼97%, thus above 95% ANI that corresponds to the 70% DNA-DNA hybridization cutoff value widely used to delineate species (Goris et al., 2007). *Shewanella* sp. Alg231_23 isolated from a sponge might be an opportunistic or transient bacterium detected during sampling. Furthermore, comparison of *Shewanella* sp. OPT22 with other *Shewanella* strains showed an average ANI value ≤ 72% (Supplementary Table S2). Evidence suggests that the sponge-associated *Shewanella* sp. OPT22 sequenced in this study could be a novel strain closely related to *S*. *irciniae*.

### General Genome Characters of Sponge-Associated *Shewanella*

**FIGURE 3:**
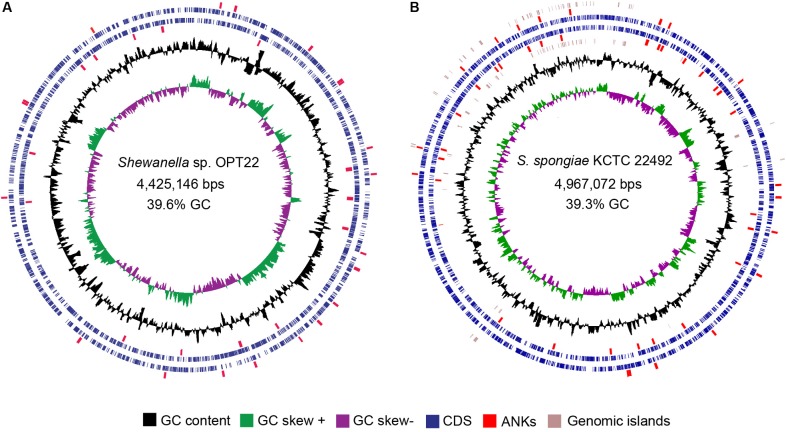
Circular view of the genomes of two sponge-associated *Shewanella* species, generated with CGview (Stothard and Wishart, 2005). The genome map of **(A)** *Shewanella* sp. OPT22 sequenced in this study, isolated from an Atlantic sponge, *O*. *papilla* **(B)** *S. spongiae* KCTC 22492 isolated from an Eastern Sea (Korea) marine sponge. Circles from interior to exterior represent GC skew and GC content (black circle). Blue circles denote the coding sequences on forward and reverse strands. GIs are represented as brown circles in the genome of *S. spongiae* KCTC 22492. The ankyrin-repeat domains containing proteins detected in the genomes are highlighted in red.

**FIGURE 4:**
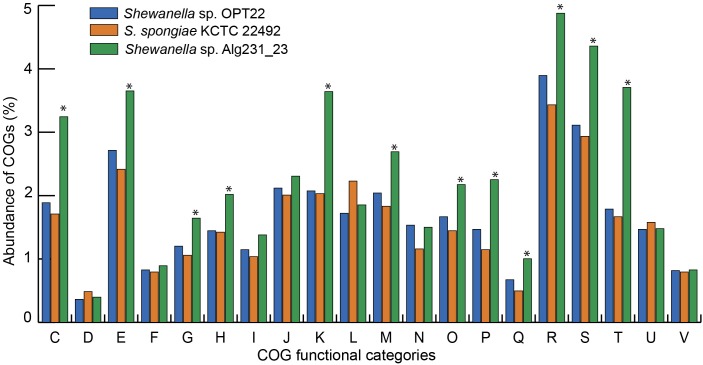
Bar graph representing the relative abundance of COGs in each of the three sponge-associated *Shewanella* species. COG functional classes from C-V were shown. *Shewanella* sp. OPT22 sequenced in this study was represented in blue and other two genomes of sponge-associated *Shewanella* species- KCTC 22492 and Alg231_23 compared in this analysis were represented in light-brown and green, respectively. The asterisks denote significant abundance of COG categories (*p* < 0.01).

The genome assembly of the strain *Shewanella* sp. OPT22 retrieved a total of 69 contigs (*N*_50_ of > 0.30 Mb) and a chromosomal genome size of 4.4 Mbp with a low G + C content of 39.6% (Figure 3A). The genome was near complete (93.6%) and had low evidence of possible contamination (0.37%) based on a battery of 519 *Alteromonadales* marker genes. The genome encoded a total of 3,971 genes, of which 3,840 (96.7%) were protein-coding and 108 were RNA-coding. The functional annotation of genes based on cluster of orthologous (COG) groups is shown in Supplementary Table S3. The detailed genome characteristic features of the three sponge-associated bacteria (*Shewanella* sp. OPT22, *S*. *spongiae* KCTC 22492, and *Shewanella* sp. Alg231_23) are given in Table 2. Comparison of COG categories among three sponge-associated *Shewanella* species revealed a significant overrepresentation of functional categories (*Z*-test, *p* < 0.01) – COG ‘C’ (Energy production and conversion), ‘E’ (Amino acid transport and metabolism), ‘G’ (Carbohydrate transport and metabolism), ‘H’ (Coenzyme transport and metabolism), ‘K’ (Transcription), ‘M’ (Cell wall/membrane/envelope biogenesis), ‘O’ (Posttranslational modification, protein turnover, chaperones), ‘P’ (Inorganic ion transport and metabolism), ‘Q’ (Secondary metabolites biosynthesis, transport and catabolism), ‘R’ (General function prediction only), ‘S’ (Function unknown), and ‘T’ (Signal transduction mechanisms) in the genome of another sponge-associated bacterium, *Shewanella* sp. Alg231_23 isolated from a sponge, *S. officinalis* (Figure 4). This trend is obvious considering a larger genome size of *Shewanella* sp. Alg231_23 (5.8 Mbp) when compared to *Shewanella* sp. OPT22 (4.4 Mbp) and *S*. *spongiae* KCTC 22492 (4.9 Mbp). Many evidences suggest the existence of trends between functional gene content and genome size (Stover et al., 2000; Jordan et al., 2001; Bentley et al., 2002), and disproportionate enrichment of the genome attributes responsible for regulation and secondary metabolism genes in larger genomes (Konstantinidis and Tiedje, 2004). Furthermore, we detected a uniform distribution of genes belonging to COG functional categories between two closely related sponge-associated bacteria: *Shewanella* sp. OPT22 and *S*. *spongiae* KCTC 22492.

**Table 2:** Genome characteristics of the three sponge-associated *Shewanella* species.

	*Shewanella* sp. OPT22	*S. spongiae* KCTC 22492	*Shewanella* sp. Alg231_23
Genome size (bp)	4,425,146	4,967,072	5,817,724
# contigs	69	318	23
Largest contig (bp)	719,047	87003	1,830,006
Contig N50	306,208	31,328	502,833
GC (%)	39.60	39.33	43.60
# Genes	3,971	4019	5,155
# CDS	3,840	3,878	4,992
COGs (%)	71.8	66.6	74.4
# rRNA	9	8	11
# tRNA	98	83	117
#ANKs	45	52	4
#TPRs	29	20	41
#Sel1	5	3	6


**Shewanella* sp. OPT22 was sequenced in this study.*

### Predicted Genomic Architecture of Sponge-Associated *Shewanella* for the Successful Association With the Marine Sponge

#### Eukaryotic-Like Proteins and Abundance of Ankyrin-Like Protein Domains

**FIGURE 5:**
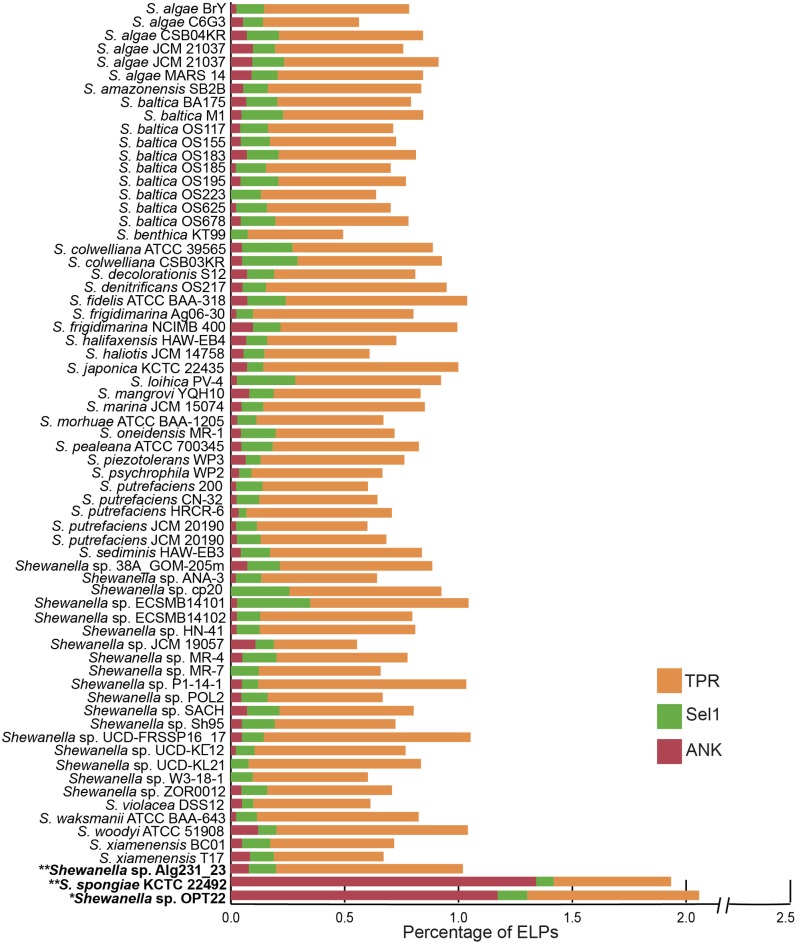
Staked bar plot showing the percentage abundance of eukaryotic-like proteins (ELPs) detected in the genus *Shewanella*. A total of 68 genomes were compared for the presence of proteins-containing ELPs- ankyrin-repeat (ANKs), tetratrico peptide repeats (TPRs), and Sel1 repeats. The strains isolated from the sponges are shown in bold. Single and double asterisks denote the genomes sequenced in this study and in previous studies, respectively.

The genome of *Shewanella* sp. OPT22 was encoded with certain ELPs containing motifs such as ANKs, TPRs, and Sel1 repeats. ELPs (symbiosis factors) have been commonly detected in pathogenic and symbiotic microbes, playing a vital role in intracellular survival and pathogenicity by interfering with the host protein-protein interactions (Habyarimana et al., 2008; Gomez-Valero et al., 2011). An abundance of proteins containing ankyrin-like domains (ANKs; *n* = 45; *p* < 0.01) was detected in the genome of sponge-associated *Shewanella* sp. OPT22 sequenced in this study when compared to other bacterial members of the genus *Shewanella* (Figure 5 and Supplementary Tables S4, S5). All the other strains encoded less than 10 ANKs (six strains were devoid of ANKs) in their genomes (Figure 5 and Supplementary Table S5). Overrepresentation of ANKs in *Shewanella* sp. OPT22 could be a key feature of the sponge-associated lifestyle of this bacterium. Previous study reported a frequent detection of ANKs in symbiotic microbes and suggested that lifestyle is a determinant factor for the abundance of ANKs (Jernigan and Bordenstein, 2014). Affirming the previous statement, the genome of another closely related bacterium- *S*. *spongiae* KCTC 22492 also harbored a high number of ANKs (*n* = 52). ANKs detected in *Shewanella* sp. OPT22 and *S*. *spongiae* KCTC 22492 might facilitate the host immune evasion and the bacterial survival inside the sponge host. Metagenomics of microbial consortium associated with a sponge *Cymbastela concentrica* (Thomas et al., 2010; Liu et al., 2012), metaproteogenomics of several sponge-associated microbes (Fan et al., 2012), and the genome analyses of symbiont-*Poribacteria* (Siegl et al., 2011), *Deltaproteobacteria* (Liu et al., 2011), and *Pseudovibrio* spp. isolated from different sponges (Bondarev et al., 2013; Alex and Antunes, 2015, 2018; Versluis et al., 2018) revealed the presence of ELPs. Abundance of ANKs in the microbiome of healthy sponges (Fan et al., 2012; Liu et al., 2012) and their loss in sponges experiencing thermal stress and bleaching (Fan et al., 2013) indicate that ANKs play a crucial role in sponge-bacteria symbioses. The role of ANKs in modulating phagocytosis by amoeba and intracellular survival was experimentally validated by heterologous expression of sponge symbiont encoded ANKs, promoting bacteria-eukaryote symbiosis (Nguyen et al., 2014).

Interestingly other ELPs, like TPRs and Sel1, were widespread in all the genomes analyzed here, independent of the lifestyle and habitat (Figure 5 and Supplementary Table S5). The genome of *Shewanella* sp. OPT22 encoded fewer proteins with TPRs (*n* = 29) and Sel1 repeats (*n* = 5). The genome of another sponge-associated bacterium, *Shewanella* sp. Alg231_23showed less number of ANKs (*n* = 4), and Sel1 repeats (*n* = 6); and an overabundance of TPRs (*n* = 41). A similar trend was detected in *S*. *woodyi* ATCC 51908, a closely related strain isolated from the squid (ANKs; *n* = 6, Sel1; *n* = 4, TPRs; *n* = 42). Our analyses (see previous section) showed that Alg231_23 is phylogenetically closer to *S*. *woodyi* ATCC 51908 than to OPT22, affirming the statement that rather than lifestyle; phylogenetic history is responsible for the abundance of TPRs (Jernigan and Bordenstein, 2015). However, the aforementioned statement should be interpreted with caution due to the limited number of the genomic data of sponge-associated *Shewanella* species (*n* = 3) currently available. Isolation of more *Shewanella* strains from sponges might give further insight of the role of ELPs in the sponge-associated *Shewanella*.

#### Role of Secretion Systems in *Shewanella*-Sponge Symbioses

**FIGURE 6:**
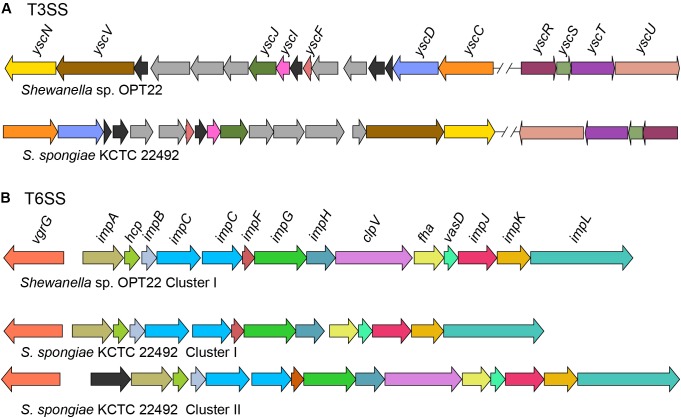
Genetic organization of major SSs in *Shewanella* sp. OPT22 sequenced in this study and *S. spongiae* KCTC 22492. **(A)** Predicted type III secretion system (T3SS) core components in the genome of OPT22 and KCTC 22492. Detected T3SS clusters are shown as cluster I and cluster II. Gene clusters detected in different contigs are separated by double cross-hatched lines. **(B)** Predicted genes coding for type VI secretion system (T6SS). One gene cluster was detected in the OPT22, whereas two clusters (cluster I and cluster II) in KCTC 22492. Different color schemes are given for each predicted genes in all the SSs. Functionally related group of genes are indicated with the same color in each SS. Black (T3SSs and T6SSs) and dark-gray (T3SS) colors denote predicted hypothetical and unrelated genes. The genes are not shown to scale.

**FIGURE 7:**
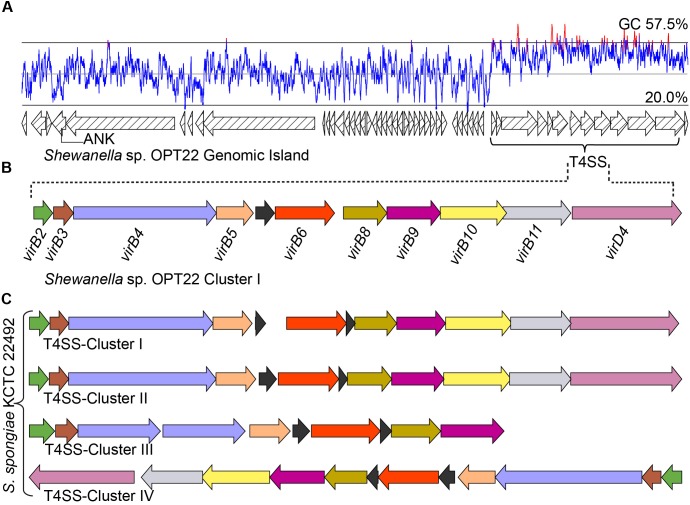
Genomic island (GI) and type IV SS. **(A)** Predicted GI encoding T4SS gene cluster in *Shewanella* sp. OPT22. The line graph above the GI represents the GC content. A gene encoding for ankyrin-repeat containing protein is also shown. **(B)** Genetic organization of the type IV SS in *Shewanella* sp. OPT22 (cluster I). **(C)** T4SS gene clusters detected in *S. spongiae* KCTC 22492 (cluster I, II, III, and IV). Different color schemes are given for each predicted SS genes. Functionally related group of genes are indicated with the same color in each SS in both species. Black color denotes predicted hypothetical genes. The genes are not shown to scale.

Symbiotic microbes utilize multitude of methods to colonize and invade eukaryotic hosts. One such mechanism involves the transport of proteins across the membrane using specialized translocation systems called secretions systems (SSs) to thwart the host immune response and facilitate the bacterial invasion (Green and Mecsas, 2016). KEGG analysis detected the genes coding for type III SS (T3SS) (M00332), type IV SS (T4SS) (M00333), and type VI SS (T6SS) (M00334) in the genome of *Shewanella* sp. OPT22 (Figure 6, 7 and Supplementary Table S6) suggesting the likely ability of the sequenced bacterial strain to export and inject the effector/virulence molecules into the host cell for the establishment of sponge-bacteria interaction. The details of each SSs are discussed below.

### Type III Secretion System

Type III secretion systems or “injectisomes” are complex machineries, that provide commensal and pathogenic bacteria with a unique virulence mechanism enabling them to inject the effector from the bacterial cytosol to the cytoplasm/ plasma membrane of the target (host) cells (Puhar and Sansonetti, 2014). In the genome of *Shewanella* sp. OPT22, we detected the genes encoding for T3SS (non-flagellar T3SS) core components (Figure 6A) and four copies of homologs of T3SS effector (T3E) molecules EspA-like secreted protein (pfam03433) and HopW1-1 (Hrp outer protein). Enteropathogenic *Escherichia coli* (EPEC) secreted protein *A* (EspA) is a hydrophilic translocon component responsible for the attachment of the bacterium to the host cell surface by forming a sheath-like filament (Knutton et al., 1998; Sekiya et al., 2001), which aids to penetrate the host mucous barrier (Daniell et al., 2001). The role of other T3Es in the disruption of actin cytoskeleton and inhibition of endocytosis was reported among the plant pathogen *Pseudomonas syringae* (Kang et al., 2014). Further analyses revealed syntenic organization of T3SS gene cluster of two sponge-associated bacteria, *Shewanella* sp. OPT22 and *S. spongiae* KCTC 22492 (Figure 6A). The genome of *S. spongiae* KCTC 22492 was also encoded with the homologs of genes responsible for T3E molecules-EspA, pathogenicity island two effector protein SseD, and PipB2 (Pathogenicity island-encoded protein). SseD and PipB2 are reported to function as virulence factors in *Salmonella*, required for establishing infection of the mouse (Klein and Jones, 2001) and maintenance of intracellular pathogenic lifestyle (Henry et al., 2006). Non-flagellar T3SS and genes encoding effector molecules were not detected in another sponge-associated bacterium-*Shewanella* sp. Alg231_23. Absence of an effective T3SS could possibly widen the range of marine host species on which *Shewanella* strains can colonize. A similar trend was observed among the plant pathogen *Pseudomonas syringae*, as an adaptation mechanism to reduce the fitness costs of host-specific virulence in the presence of other ephemeral host plants (Kniskern et al., 2011).

### Type VI Secretion System

Type VI secretion systems (T6SS), are widespread in Gram-negative bacteria and have been shown to be involved in pathogenicity, inter- and intra-species competition, bacterial communication (quorum sensing), and biofilm formation (Alteri and Mobley, 2016; Dang and Lovell, 2016; Gallique et al., 2017). In the genome of *Shewanella* sp. OPT22, we detected a 21 Kbp T6SS gene cluster that encodes genes that are predicted to the 13 core T6SS proteins (Figure 6B and Supplementary Table S6). In the case of *S. spongiae* KCTC 22492, the T6SS-associated genes are located in two clusters with different sizes-T6SS-I (18 Kbp) and T6SS-II (23 Kbp) (Figure 6B). The prevalence of extra gene cluster coding for T6SS might be advantageous for unknown function, as suggested in *Pantoea ananatis* strains inhabiting different environments (Shyntum et al., 2014). Both T6SS-I and II clusters of *S. spongiae* KCTC 22492 encoded the core T6SS proteins, which if expressed could produce the complete machinery for T6SS. Further experimental validation is required to test whether T6SS encoded genes are transcribed in both the clusters. Moreover, the genetic organization of T6SS gene cluster is conserved in *Shewanella* sp. OPT22 and *S. spongiae* KCTC 22492. Predicted T6SS gene clusters in the genomes of two sponge-associated *Shewanella* spp., also encoded the *hcpI* (COG3157) and *vgrG* (COG3501) genes with possible effector functions (Bönemann et al., 2010). Homologs of *hcpI* and *vgrG* in multiple copies were identified in the genomes of sponge-associated *Pseudovibrio* spp. (Bondarev et al., 2013; Alex and Antunes, 2015, 2018; Romano et al., 2016). However, we did not identify extra copies of T6SS effector molecules- *hcpI* and *vgrG* in the genomes of both sponge-associated *Shewanella* species. Analysis of *Shewanella* sp. Alg231_23, isolated from a sponge, revealed syntenic genetic organization of T6SS gene cluster with closely related *S*. *woodyi* ATCC 51908 (Supplementary Figure S2). We assume that T6SSs of both *Shewanella* species are functional, due to the presence of all structural components of the T6SS apparatus that presumably act as a virulence factor by delivering the effector proteins into the sponge host cell.

### T4SS and Localization of ANKs Within the GIs of Sponge-Associated *Shewanella*

In addition to the T3 and T6SSs, we identified a T4SS in the genome of *Shewanella* sp. OPT22 (Figure 7A,B and Supplementary Table S7). T4SS is homologous to bacterial conjugation system and perform pivotal biological functions, namely DNA exchange mediating horizontal gene transfer (HGT) and translocation of proteins, protein-DNA complexes, effector/virulence molecules into the target cells (Wallden et al., 2010). T4SSs loci of *Shewanella* sp. OPT22 encoded the homologs of virulence genes: *virB2* (K03197), *virB3* (K03198), *virB4* (K03199), *virB5* (K03200), *virB6* (K03201), *virB8* (K03203), *virB9* (K03204), *virB10* (K03195), *virB11* (K03196), and *virD4* (K03205) (Figure 7B). These VirB proteins synthesized from the *virB* operon, functions to form the pilus and translocation channels spanning the cell envelope of bacteria (Voth et al., 2012). It may also contribute significantly to the development of pathogenesis/symbiosis by promoting the surface adhesion, colonization, and biofilm formation.

Interestingly, T4SS was encoded in a 45 Kbp GI in the chromosomal genome of *Shewanella* sp. OPT22 (Figure 7A,B and Supplementary Table S7) suggesting that the identified T4SS belong to GI type or GI-associated T4SS and possible role of T4SS in the dissemination of GIs in sponge-associated *Shewanella* spp. We detected the GIs in all three sponge-associated *Shewanella* species (Supplementary Figure S3). These self transmissible GIs are capable of excision, replication, conjugal transfer, replication, and integration into recipient bacterial chromosome. In addition, GI-T4SSs also carry many cargo genes, apparently not related/essential for the conjugative transfer, but are interspersed within the GIs (Johnson and Grossman, 2015). The role of T4SSs in the mobilization of GIs, enabling the dissemination of cargo genes namely, antibiotic resistance or virulence genes and catabolic genes, was reported among pathogenic bacteria *Haemophilus influenza* (Juhas et al., 2007) and *Pseudomonas* sp. strain B13 (Gaillard et al., 2006). Most of the GI encoded genes of *Shewanella* sp. OPT22 were predicted as hypothetical. Strikingly, one gene encoding for proteins containing ankyrin-like domain was detected within the GI (Figure 7A). The detected ANK-containing protein lacked signal sequence for general SS pathway; GI-encoded ANK could be a possible candidate for T4SS effector molecule and may be involved in the modulation of host-cell functions (Nakayama et al., 2008). Our subcellular localization analyses of this particular GI, detected a higher number of genes belonging to “unknown” category. It is noteworthy that more genes in GI with “unknown” subcellular localization indicate the absence of orthologous matches in the database, suggesting the presence of the novel/uncharacterized genes or genes acquired recently through the horizontal gene transfer (Cortez et al., 2009). A similar trend was detected in the genomes of the members of the genus *Pseudovibrio* isolated from different marine invertebrate hosts (Alex and Antunes, 2018).

The genome of *S. spongiae* KCTC 22492, another sponge-associated bacterium carried multiple T4SS gene clusters, with ten or more genes per cluster (Figure 7C). Genomes of obligate intracellular *Rickettsia* spp. were reported to carry multiple T4SS gene clusters flanked by integrase or tRNA genes, likely representing the remnants of ancient GIs (Cho et al., 2007). However, we did not observe integrase or tRNA genes flanking the T4SS gene clusters in the genome of *S. spongiae* KCTC 22492. Though, GI type T4SS was not detected, the GIs of *S. spongiae* KCTC 22492 harbored seven genes encoding for ankyrin-like domains (Figure 3B and Supplementary Table S8). Enrichment of genes coding for ANKs involved in host-parasite interaction has been reported in the GIs of obligate intracellular parasites-*Babela massiliensis* (lives in *Acanthamoeba castellaniiparasitic*) (Pagnier et al., 2015), *Orientia tsutsugamushi* (lives in trombiculid mites) (Nakayama et al., 2008), and in a bacterium, *Burkholderia cenocepacia*, for successful pathogenic lifestyle (Patil et al., 2017). The detection of ANks within the GIs of both sponge-associated *Shewanella* spp. clearly suggest the procurement of certain adaptive traits such as evasion of the bacteria against the host immunity, and thus enhance the chances of survival within the sponge hosts. However, the GIs of another sponge-associated bacterium, *Shewanella* sp. Alg231_23 did not encode any predicted ANKs, indicating the possible procurement of some of the ANKs through HGT in the sponge-associated bacteria, *Shewanella* sp. OPT22 and *S. spongiae* KCTC 22492.

## Conclusion

In this study, the genomic features of sponge-associated *Shewanella* were investigated by comparing them with the genomes of other members of the genus *Shewanella* isolated from different habitats. The phylogenetic clustering of a sponge-associated *Shewanella* sp. OPT22 sequenced in this study and previously reported sponge bacterium *S. spongiae* KCTC 22492 with the 16S rRNA sequences of bacteria isolated from geographically separated different sponges (e.g., Atlantic vs. Pacific - East Sea – regions) reveals the “sponge-specific clustering” of these two strains of *Shewanella*. Comparative genomic analyses with other members of the genus *Shewanella* showed an overabundance of the ankyrin-repeat domains containing proteins (ANKs) in the genome of *Shewanella* sp. OPT22 and *S*. *spongiae* KCTC 22492, a key genomic feature which might interfere with phagocytosis and facilitate the bacterium to evade the digestion in a sponge host. Moreover, the major SSs machineries detected in the *Shewanella* sp. OPT22 and *S*. *spongiae* KCTC 22492 might be involved in delivering the lethal effector proteins across the bacterial membrane to the target cell in order to gain control over the host system. Localization of T4SS on a GI of *Shewanella* sp. OPT22 and cargo genes, particularly the ANKs in both *Shewanella* sp. OPT22 and *S*. *spongiae* KCTC 22492, suggest the possible role of horizontal gene transfer that might contribute to the evolution of sponge-associated *Shewanella* species and its adaptation to sponge-specific niches. Conclusively, the genome of true symbiotic *Shewanella* strains isolated from sponges described here has the genomic signatures for symbiotic lifestyle and may further offer insights into the molecular strategies for niche-specific lifestyle.

## Data Deposition

This project has been deposited at GenBank under the accession number PGVH00000000.

## Author Contributions

AAn analyzed the data, contributed the reagents, and drafted the manuscript. AAl designed the work, executed the experiments, analyzed the data, and drafted the manuscript.

## Conflict of Interest Statement

The authors declare that the research was conducted in the absence of any commercial or financial relationships that could be construed as a potential conflict of interest.

## Footnotes

1 ftp://ftp.ncbi.nih.gov/pub/mmdb/cdd/little_endian/, accessed on June 2017

2 https://github.com/kblin/ncbi-genome-download, accessed on June 2017
