# Supplementary material for: Whole-Genome Comparisons Among the Genus Shewanella Reveal the Enrichment of Genes Encoding Ankyrin-Repeats Containing Proteins in Sponge-Associated Bacteria
Source: Front Microbiol. 2019 Feb 6;10:5. doi: 10.3389/fmicb.2019.00005 (PMC6372511; doi:10.3389/fmicb.2019.00005)
Supplement: Supplementary file 1 [file Table_1.DOCX]

| **Table S1. List of organisms used for comparative genomics** | |  |  |  |  |
| --- | --- | --- | --- | --- | --- |
| **#Organism/Name** | **BioSample Accession** | **BioProject Accession** | **Assebly Version** | **Genome Size (Mbp)** | **GC content (%)** |
| *Shewanella algae BrY* | SAMN05580110 | PRJNA339142 | GCA_001870495.1 | 4,54712 | 52,4 |
| *Shewanella algae* C6G3 | SAMN02921234 | PRJNA255462 | GCA_000956365.1 | 4,87943 | 53,1 |
| *Shewanella algae* CSB04KR | SAMN05384438 | PRJNA328548 | GCA_001858195.1 | 4,80336 | 53,0 |
| *Shewanella algae* JCM 21037 = NBRC 103173 | SAMD00015726 | PRJDB633 | GCA_000615045.1 | 4,82769 | 53,0 |
| *Shewanella algae* JCM 21037 = NBRC 103173 | SAMD00046904 | PRJDB416 | GCA_001598875.1 | 4,81725 | 53,1 |
| *Shewanella algae* MARS 14 | SAMEA3221090 | PRJEB8326 | GCA_000947195.1 | 5,00585 | 52,9 |
| *Shewanella amazonensis* SB2B | SAMN02598298 | PRJNA13385 | GCA_000015245.1 | 4,30614 | 53,6 |
| *Shewanella baltica* BA175 | SAMN00016994 | PRJNA49719 | GCA_000147735.3 | 5,1994 | 46,1 |
| *Shewanella baltica* M1 | SAMN04555237 | PRJNA315288 | GCA_001620325.1 | 5,16758 | 46,2 |
| *Shewanella baltica* OS117 | SAMN00713637 | PRJNA49451 | GCA_000215895.1 | 5,52602 | 46,0 |
| *Shewanella baltica* OS155 | SAMN02598299 | PRJNA13386 | GCA_000015845.1 | 5,3429 | 46,2 |
| *Shewanella baltica* OS183 | SAMN02256524 | PRJNA50319 | GCA_000179535.2 | 5,04993 | 46,1 |
| *Shewanella baltica* OS185 | SAMN02598398 | PRJNA17643 | GCA_000017325.1 | 5,31291 | 46,3 |
| *Shewanella baltica* OS195 | SAMN00623061 | PRJNA13389 | GCA_000018765.1 | 5,54754 | 46,2 |
| *Shewanella baltica* OS223 | SAMN00623062 | PRJNA17985 | GCA_000021665.1 | 5,35888 | 46,3 |
| Shewanella baltica OS625 | SAMN02256537 | PRJNA60225 | GCA_000231345.2 | 5,19524 | 46,3 |
| *Shewanella baltica* OS678 | SAMN00016780 | PRJNA47019 | GCA_000178875.2 | 5,36877 | 46,3 |
| *Shewanella benthica* KT99 | SAMN02436096 | PRJNA13387 | GCA_000172075.1 | 4,35159 | 46,0 |
| *Shewanella colwelliana* ATCC 39565 | SAMN02584968 | PRJNA204100 | GCA_000518705.1 | 4,57562 | 45,4 |
| *Shewanella colwelliana* CSB03KR | SAMN05384437 | PRJNA328548 | GCA_001735525.1 | 4,64254 | 45,3 |
| *Shewanella decolorationis* S12 | SAMN02469853 | PRJNA210345 | GCA_000485795.1 | 4,84397 | 47,1 |
| *Shewanella denitrificans* OS217 | SAMN02598300 | PRJNA13390 | GCA_000013765.1 | 4,54591 | 45,1 |
| *Shewanella fidelis* ATCC BAA-318 | SAMN02584974 | PRJNA204111 | GCA_000518605.1 | 4,79869 | 42,8 |
| *Shewanella frigidimarina* Ag06-30 | SAMN04413751 | PRJNA308795 | GCA_001529365.1 | 4,79663 | 41,2 |
| *Shewanella frigidimarina* NCIMB 400 | SAMN02598301 | PRJNA13391 | GCA_000014705.1 | 4,84526 | 41,6 |
| Shewanella halifaxensis HAW-EB4 | SAMN02598431 | PRJNA20241 | GCA_000019185.1 | 5,22692 | 44,6 |
| Shewanella haliotis JCM 14758 | SAMD00004655 | PRJDB630 | GCA_000614935.1 | 4,92992 | 52,9 |
| *Shewanella japonica* KCTC 22435 | SAMN06628905 | PRJNA380174 | GCA_002075795.1 | 4,97568 | 40,8 |
| *Shewanella loihica* PV-4 | SAMN00623064 | PRJNA13906 | GCA_000016065.1 | 4,60259 | 53,7 |
| *Shewanella mangrovi* YQH10 | SAMN02887322 | PRJNA253757 | GCA_000753795.1 | 4,21579 | 48,1 |
| *Shewanella marina* JCM 15074 | SAMD00011086 | PRJDB631 | GCA_000614975.1 | 4,42465 | 40,4 |
| *Shewanella morhuae* ATCC BAA-1205 | SAMN05421840 | PRJEB18895 | GCA_900156405.1 | 4,19037 | 44,0 |
| *Shewanella oneidensis* MR-1 | SAMN02604014 | PRJNA335 | GCA_000146165.2 | 5,13142 | 45,9 |
| *Shewanella pealeana* ATCC 700345 | SAMN02598386 | PRJNA17415 | GCA_000018285.1 | 5,17458 | 44,7 |
| *Shewanella piezotolerans* WP3 | SAMN02603456 | PRJNA17675 | GCA_000014885.1 | 5,39648 | 43,3 |
| *Shewanella psychrophila* WP2 | SAMN04370084 | PRJNA306925 | GCA_002005305.1 | 6,35341 | 44,3 |
| *Shewanella putrefaciens* 200 | SAMN00632276 | PRJNA13392 | GCA_000169215.2 | 4,84025 | 44,5 |
| *Shewanella putrefaciens* CN-32 | SAMN00623063 | PRJNA13393 | GCA_000016585.1 | 4,65922 | 44,5 |
| *Shewanella putrefaciens* HRCR-6 | SAMN02584939 | PRJNA195875 | GCA_000519065.1 | 3,63135 | 45,4 |
| *Shewanella putrefaciens* JCM 20190 = NBRC 3908 | SAMD00046716 | PRJDB463 | GCA_001591325.1 | 4,33612 | 44,3 |
| *Shewanella putrefaciens* JCM 20190 = NBRC 3908 | SAMD00004020 | PRJDB632 | GCA_000615005.1 | 4,32798 | 44,3 |
| *Shewanella sediminis* HAW-EB3 | SAMN02598412 | PRJNA18789 | GCA_000018025.1 | 5,51767 | 46,1 |
| *Shewanella spongiae* KCTC 22492 | SAMN10081109 | PRJNA491421 | NA | 4,96707 | 39,3 |
| *Shewanella* sp. 38A_GOM-205m | SAMN02584982 | PRJNA213750 | GCA_000518445.1 | 4,73935 | 53,1 |
| *Shewanella* sp. Alg231_23 | SAMEA3925393 | PRJEB13410 | GCA_900079515.1 | 5,81772 | 43,6 |
| *Shewanella* sp. ANA-3 | SAMN02598318 | PRJNA13905 | GCA_000203935.1 | 5,25115 | 48,0 |
| *Shewanella* sp. cp20 | SAMN02904642 | PRJNA242566 | GCA_000832025.1 | 4,47455 | 53,1 |
| *Shewanella* sp. ECSMB14101 | SAMN03140421 | PRJNA264758 | GCA_000773485.1 | 4,26807 | 49,8 |
| *Shewanella* sp. ECSMB14102 | SAMN03252485 | PRJNA269200 | GCA_000813075.1 | 4,41244 | 52,2 |
| *Shewanella* sp. HN-41 | SAMN02469863 | PRJNA66317 | GCA_000217915.2 | 4,41403 | 46,5 |
| *Shewanella* sp. JCM 19057 | SAMD00000388 | PRJDB1629 | GCA_001310535.1 | 4,89958 | 53,1 |
| *Shewanella* sp. MR-4 | SAMN02598317 | PRJNA13904 | GCA_000014685.1 | 4,70629 | 47,9 |
| *Shewanella* sp. MR-7 | SAMN02598316 | PRJNA13903 | GCA_000014665.1 | 4,79911 | 47,9 |
| *Shewanella* sp. P1-14-1 | SAMN04043846 | PRJNA294442 | GCA_001401775.1 | 4,91642 | 40,8 |
| *Shewanella* sp. POL2 | SAMN02469948 | PRJNA169039 | GCA_000282755.1 | 4,91395 | 46,2 |
| *Shewanella* sp. SACH | SAMN05990718 | PRJNA352664 | GCA_001887095.1 | 4,92942 | 46,5 |
| *Shewanella* sp. Sh95 | SAMN03892644 | PRJNA290501 | GCA_001308045.1 | 4,82087 | 46,3 |
| *Shewanella* sp. UCD-FRSSP16_17 | SAMN05172614 | PRJNA322705 | GCA_001675935.1 | 4,96018 | 40,7 |
| *Shewanella* sp. UCD-KL12 | SAMN06007749 | PRJNA353005 | GCA_001957125.1 | 5,69153 | 43,2 |
| *Shewanella* sp. UCD-KL21 | SAMN06007764 | PRJNA353007 | GCA_001957135.1 | 4,59877 | 42,0 |
| *Shewanella* sp. W3-18-1 | SAMN02598315 | PRJNA13902 | GCA_000015185.1 | 4,70838 | 44,6 |
| *Shewanella* sp. ZOR0012 | SAMN03021526 | PRJNA205575 | GCA_000798835.1 | 4,9532 | 46,0 |
| *Shewanella violacea* DSS12 | SAMD00060963 | PRJDA34739 | GCA_000091325.1 | 4,9621 | 44,7 |
| *Shewanella waksmanii* ATCC BAA-643 | SAMN02584962 | PRJNA204050 | GCA_000518805.1 | 4,97148 | 45,3 |
| *Shewanella woodyi* ATCC 51908 | SAMN02598391 | PRJNA17455 | GCA_000019525.1 | 5,9354 | 43,7 |
| *Shewanella xiamenensis* BC01 | SAMN02678192 | PRJNA238517 | GCA_000712635.2 | 4,67259 | 46,2 |
| *Shewanella xiamenensis* T17 | SAMN03704056 | PRJNA284612 | GCA_001723195.1 | 5,36819 | 46,3 |
