# Supplementary material for: Whole-Genome Comparisons Among the Genus Shewanella Reveal the Enrichment of Genes Encoding Ankyrin-Repeats Containing Proteins in Sponge-Associated Bacteria
Source: Front Microbiol. 2019 Feb 6;10:5. doi: 10.3389/fmicb.2019.00005 (PMC6372511; doi:10.3389/fmicb.2019.00005)
Supplement: Supplementary file 3 [file Table_3.DOCX]

**Table S3**. The distribution of protein-coding genes of *Shewanella* sp. OPT22 into cluster of orthologous group (COG) categories

| **COG categories** |  |
| --- | --- |
| [A] RNA processing and modification | 1 |
| [B] Chromatin structure and dynamics | 1 |
| [C] Energy production and conversion | 171 |
| [D] Cell cycle control, cell division, chromosome partitioning | 33 |
| [E] Amino acid transport and metabolism | 246 |
| [F] Nucleotide transport and metabolism | 75 |
| [G] Carbohydrate transport and metabolism | 109 |
| [H] Coenzyme transport and metabolism | 131 |
| [I] Lipid transport and metabolism | 104 |
| [J] Translation, ribosomal structure and biogenesis | 192 |
| [K] Transcription | 188 |
| [L] Replication, recombination and repair | 156 |
| [M] Cell wall/membrane/envelope biogenesis | 185 |
| [N] Cell motility | 139 |
| [O] Posttranslational modification, protein turnover, chaperones | 151 |
| [P] Inorganic ion transport and metabolism | 133 |
| [Q] Secondary metabolites biosynthesis, transport and catabolism | 61 |
| [R] General function prediction only | 353 |
| [S] Function unknown | 282 |
| [T] Signal transduction mechanisms | 162 |
| [U] Intracellular trafficking, secretion, and vesicular transport | 133 |
| [V] Defense mechanisms | 74 |
