# Supplementary material for: Whole-Genome Comparisons Among the Genus Shewanella Reveal the Enrichment of Genes Encoding Ankyrin-Repeats Containing Proteins in Sponge-Associated Bacteria
Source: Front Microbiol. 2019 Feb 6;10:5. doi: 10.3389/fmicb.2019.00005 (PMC6372511; doi:10.3389/fmicb.2019.00005)
Supplement: Supplementary file 4 [file Table_4.DOCX]

**Table S4**. List of predicted proteins coding for eukaryotic-like domains – ankyrin repeats (ANKs), tetratrico peptide repeats (TPRs), and Sel1 repeats in the genome of *Shewanella* sp. OPT22

| **Locus ID** | **Product** | **Interpro ID** |
| --- | --- | --- |
|  | **ORFs containing ANK domains** |  |
| SOPP22_00093 | Ankyrin repeats (3 copies) | IPR020683 |
| SOPP22_00140 | Ankyrin repeats (3 copies) | IPR020683 |
| SOPP22_00266 | Ankyrin repeats (3 copies) | IPR002110 |
| SOPP22_00448 | hypothetical protein | IPR020683 |
| SOPP22_00593 | Ankyrin repeats (3 copies) | IPR002110 |
| SOPP22_00598 | Ankyrin repeats (many copies) | IPR002110 |
| SOPP22_00721 | hypothetical protein | IPR020683 |
| SOPP22_00832 | Ankyrin repeats (3 copies) | IPR020683 |
| SOPP22_00890 | Ankyrin repeat protein | IPR002110 |
| SOPP22_00893 | Ankyrin repeats (3 copies) | IPR020683 |
| SOPP22_00918 | hypothetical protein | IPR002110 |
| SOPP22_00991 | Ankyrin repeats (3 copies) | IPR020683 |
| SOPP22_01080 | hypothetical protein | IPR020683 |
| SOPP22_01083 | Ankyrin repeats (3 copies) | IPR020683 |
| SOPP22_01165 | Ankyrin repeats (3 copies) | IPR020683 |
| SOPP22_01205 | Ankyrin repeats (3 copies) | IPR002110 |
| SOPP22_01206 | Ankyrin repeats (many copies) | IPR020683 |
| SOPP22_01362 | hypothetical protein | IPR020683 |
| SOPP22_01459 | Phosphocholine transferase AnkX | IPR002110 |
| SOPP22_01460 | Phosphocholine transferase AnkX | IPR020683 |
| SOPP22_01461 | hypothetical protein | IPR020683 |
| SOPP22_01515 | Ankyrin repeats (3 copies) | IPR020683 |
| SOPP22_01557 | Phosphocholine transferase AnkX | IPR020683 |
| SOPP22_01686 | Ankyrin repeats (many copies) | IPR002110 |
| SOPP22_01708 | Ankyrin repeats (many copies) | IPR002110 |
| SOPP22_01709 | Ankyrin repeats (3 copies) | IPR020683 |
| SOPP22_01797 | Ankyrin repeats (3 copies) | IPR020683 |
| SOPP22_01951 | Ankyrin repeats (3 copies) | IPR020683 |
| SOPP22_02012 | Ankyrin repeats (many copies) | IPR020683 |
| SOPP22_02206 | Ankyrin repeats (3 copies) | IPR020683 |
| SOPP22_02354 | Ankyrin repeats (3 copies) | IPR020683 |
| SOPP22_02878 | Ankyrin repeats (3 copies) | IPR020683 |
| SOPP22_02898 | Phosphocholine transferase AnkX | IPR020683 |
| SOPP22_03080 | Ankyrin repeats (3 copies) | IPR020683 |
| SOPP22_03081 | Ankyrin repeats (3 copies) | IPR020683 |
| SOPP22_03256 | Ankyrin repeats (3 copies) | IPR002110 |
| SOPP22_03258 | hypothetical protein | IPR020683 |
| SOPP22_03262 | Phosphocholine transferase AnkX | IPR020683 |
| SOPP22_03263 | hypothetical protein | IPR020683 |
| SOPP22_03264 | Ankyrin repeat protein | IPR002110 |
| SOPP22_03524 | hypothetical protein | IPR020683 |
| SOPP22_03579 | hypothetical protein | IPR020683 |
| SOPP22_03621 | Actin-binding protein | IPR020683 |
| SOPP22_03750 | Ankyrin repeats (3 copies) | IPR020683 |
| SOPP22_03866 | Ankyrin repeats (3 copies) | IPR002110 |
|  |  |  |
|  | **ORFs containing TPR domains** |  |
| SOPP22_00395 | Hypothetical protein | - |
| SOPP22_00446 | Tetratricopeptide repeat protein | - |
| SOPP22_00468 | Tetratricopeptide repeat protein | - |
| SOPP22_00532 | Tetratricopeptide repeat protein | - |
| SOPP22_00702 | Lipoprotein NlpI | - |
| SOPP22_00737 | Response regulator SaeR | - |
| SOPP22_00795 | Beta-barrel assembly-enhancing protease | - |
| SOPP22_00825 | Tetratricopeptide repeat protein | - |
| SOPP22_00862 | type III secretion low calcium response chaperone LcrH/SycD | - |
| SOPP22_00935 | Tetratricopeptide repeat protein | - |
| SOPP22_01011 | Hypothetical protein | - |
| SOPP22_01055 | Thioredoxin | - |
| SOPP22_01210 | Chaperone protein IpgC | - |
| SOPP22_01230 | Hypothetical protein | - |
| SOPP22_01253 | Hypothetical protein | - |
| SOPP22_01415 | Formate-dependent nitrite reductase complex subunit NrfG | - |
| SOPP22_01945 | Outer membrane protein assembly factor BamD | - |
| SOPP22_02041 | Phytochrome-like protein cph2 | - |
| SOPP22_02051 | Tetratricopeptide repeat-like domain protein | - |
| SOPP22_02055 | Tetratricopeptide repeat protein | - |
| SOPP22_02258 | von Willebrand factor type A domain protein | - |
| SOPP22_02347 | Cell division coordinator CpoB | - |
| SOPP22_02383 | Beta-barrel assembly-enhancing protease | - |
| SOPP22_02621 | Beta-barrel assembly-enhancing protease | - |
| SOPP22_02775 | HTH-type transcriptional regulator MalT | - |
| SOPP22_02825 | Lipopolysaccharide assembly protein B | - |
| SOPP22_03179 | Tetratricopeptide repeat protein | - |
| SOPP22_03603 | Transcriptional regulator HilA | - |
| SOPP22_03676 | Transglutaminase-like superfamily protein | - |
|  |  |  |
|  | **ORFs containing Sel1 domains** |  |
| SOPP22_00751 | Secretory immunoglobulin A-binding protein EsiB | - |
| SOPP22_00976 | Dipeptidyl aminopeptidase BIII | - |
| SOPP22_01000 | Sel1 repeat protein | - |
| SOPP22_02518 | Hypothetical protein | - |
| SOPP22_02524 | Sel1 repeat protein | - |
