# Supplementary material for: Whole-Genome Comparisons Among the Genus Shewanella Reveal the Enrichment of Genes Encoding Ankyrin-Repeats Containing Proteins in Sponge-Associated Bacteria
Source: Front Microbiol. 2019 Feb 6;10:5. doi: 10.3389/fmicb.2019.00005 (PMC6372511; doi:10.3389/fmicb.2019.00005)
Supplement: Supplementary file 6 [file Table_6.DOCX]

**Table S6**. Genes predicted to code for type III, IV, and VI secretion system in the genome of *Shewanella* sp. OPT22

| **Locus ID** | **Product** | **Gene** | **KEGG Number** |
| --- | --- | --- | --- |
|  | **Type III secretion system- Core apparatus** |  |  |
| SOPP22_03911 | putative secretion system apparatus ATP synthase SsaN | *yscN* | K03224 |
| SOPP22_03912 | Secretion system apparatus protein SsaV | *yscV* | K03230 |
| SOPP22_03913 | hypothetical protein |  | - |
| SOPP22_03914 | HrpJ-like domain protein |  | - |
| SOPP22_03915 | type III secretion apparatus protein, HrpE/YscL family |  | - |
| SOPP22_03916 | Type III secretion system subunit |  | - |
| SOPP22_03917 | Yop proteins translocation lipoprotein J | *yscJ* | K03222 |
| SOPP22_03918 | Type III secretion basal body protein I, YscI HrpB, PscI | *yscI* | - |
| SOPP22_03919 | hypothetical protein |  | - |
| SOPP22_03920 | Type III secretion needle MxiH, YscF, SsaG,EprI, PscF, EscF | *yscF* | K03221 |
| SOPP22_03921 | Virulence regulon transcriptional activator Virf | *virF* | - |
| SOPP22_03922 | Secreted effector protein SseB | *sseB* | - |
| SOPP22_03923 | hypothetical protein |  | - |
| SOPP22_03924 | hypothetical protein |  | - |
| SOPP22_03925 | Inner membrane component of T3SS, periplasmic domain | *yscD* | K03220 |
| SOPP22_03926 | Type III secretion system outer membrane protein SpiA | *spiA/yscC* | K03219 |
|  |  |  | - |
| SOPP22_00643 | Flagellar biosynthetic protein FliP | *yscR* | K03226 |
| SOPP22_00644 | Bacterial export proteins, family 3 | *yscS* | K03227 |
| SOPP22_00645 | Bacterial export proteins, family 1 | *yscT* | K03228 |
| SOPP22_00646 | Yop proteins translocation protein U | *yscU* | K03229 |
|  |  |  |  |
|  | **Type IV secretion system** |  |  |
| SOPP22_00301 | TrbC/VIRB2 family protein | *virB2* | K03197 |
| SOPP22_00302 | Type IV secretion system protein virB3 | *virB3* | K03198 |
| SOPP22_00303 | Type IV secretion system protein virB4 | *virB4* | K03199 |
| SOPP22_00304 | Type IV secretion system protein virB5 | *virB5* | K03200 |
| SOPP22_00305 | hypothetical protein |  | - |
| SOPP22_00306 | Type IV secretion system protein VirB6 | *virB6* | K03201 |
| SOPP22_00307 | Type IV secretion system protein virB8 | *virB8* | K03203 |
| SOPP22_00308 | Type IV secretion system protein virB9 | *virB9* | K03204 |
| SOPP22_00309 | Type IV secretion system protein virB10 | *virB10* | K03195 |
| SOPP22_00310 | Type IV secretion system protein VirB11 | *virB11* | K03196 |
| SOPP22_00311 | Conjugal transfer protein TraG | *traG/virD4* | K03205 |
| SOPP22_03879 | Thiol:disulfide interchange protein DsbC | *dsbC* | K03981 |
|  |  |  |  |
|  | **Type VI secretion system** |  |  |
| SOPP22_02693 | Actin cross-linking toxin VgrG1 | *vgrG* | K11904 |
| SOPP22_02694 | ImpA, N-terminal, type VI secretion system | *impA* | K11902 |
| SOPP22_02695 | Type VI secretion system effector, Hcp | *hcp* | K11903 |
| SOPP22_02696 | Type VI secretion system, VipA, or Hcp2 | *impB* | K11901 |
| SOPP22_02697 | Type VI secretion protein, EvpB/VC_A0108, tail sheath | *impC* | K11900 |
| SOPP22_02698 | Type VI secretion protein, EvpB/VC_A0108, tail sheath |  | - |
| SOPP22_02699 | Gene 25-like lysozyme | *impF* | K11897 |
| SOPP22_02700 | Type VI secretion system, TssF | *impG* | K11896 |
| SOPP22_02701 | Type VI secretion, TssG | *impH* | K11895 |
| SOPP22_02702 | Chaperone protein ClpB 1 | *clpV* | K11907 |
| SOPP22_02703 | FHA domain protein |  | - |
| SOPP22_02704 | Type VI secretion lipoprotein, VasD, EvfM,TssJ | *vasD* | K11906 |
| SOPP22_02705 | Bacterial Type VI secretion, EvfL, ImpJ, VasE | *impJ* | K11893 |
| SOPP22_02706 | Type VI secretion system protein DotU | *impK* | K11892 |
| SOPP22_02707 | ImcF-related N-terminal domain | *impL* | K11891 |
