# Supplementary material for: Whole-Genome Comparisons Among the Genus Shewanella Reveal the Enrichment of Genes Encoding Ankyrin-Repeats Containing Proteins in Sponge-Associated Bacteria
Source: Front Microbiol. 2019 Feb 6;10:5. doi: 10.3389/fmicb.2019.00005 (PMC6372511; doi:10.3389/fmicb.2019.00005)
Supplement: Supplementary file 7 [file Table_7.DOCX]

**Table S7**. Detected genomic islands in the genome of *Shewanella* sp. OPT22. Grey shaded rows represent predicted type IV secretion system genes.

| **Locus ID** | **Product** |
| --- | --- |
| SOPP22_00263 | hypothetical protein |
| SOPP22_00264 | hypothetical protein |
| SOPP22_00265 | Integrase core domain protein |
| SOPP22_00266 | Ankyrin repeats (3 copies) |
| SOPP22_00267 | tRNA3(Ser)-specific nuclease WapA |
| SOPP22_00268 | hypothetical protein |
| SOPP22_00269 | hypothetical protein |
| SOPP22_00270 | hypothetical protein |
| SOPP22_00271 | tRNA3(Ser)-specific nuclease WapA |
| SOPP22_00272 | hypothetical protein |
| SOPP22_00273 | hypothetical protein |
| SOPP22_00274 | hypothetical protein |
| SOPP22_00275 | hypothetical protein |
| SOPP22_00276 | hypothetical protein |
| SOPP22_00277 | hypothetical protein |
| SOPP22_00278 | hypothetical protein |
| SOPP22_00279 | hypothetical protein |
| SOPP22_00280 | hypothetical protein |
| SOPP22_00281 | hypothetical protein |
| SOPP22_00282 | hypothetical protein |
| SOPP22_00283 | hypothetical protein |
| SOPP22_00284 | hypothetical protein |
| SOPP22_00285 | hypothetical protein |
| SOPP22_00286 | hypothetical protein |
| SOPP22_00287 | hypothetical protein |
| SOPP22_00288 | hypothetical protein |
| SOPP22_00289 | hypothetical protein |
| SOPP22_00290 | hypothetical protein |
| SOPP22_00291 | hypothetical protein |
| SOPP22_00292 | hypothetical protein |
| SOPP22_00293 | hypothetical protein |
| SOPP22_00294 | hypothetical protein |
| SOPP22_00295 | hypothetical protein |
| SOPP22_00296 | hypothetical protein |
| SOPP22_00297 | hypothetical protein |
| SOPP22_00298 | hypothetical protein |
| SOPP22_00299 | hypothetical protein |
| SOPP22_00300 | hypothetical protein |
| SOPP22_00301 | TrbC/VIRB2 family protein |
| SOPP22_00302 | Type IV secretion system protein virB3 |
| SOPP22_00303 | Type IV secretion system protein virB4 |
| SOPP22_00304 | Type IV secretion system protein virB5 |
| SOPP22_00305 | hypothetical protein |
| SOPP22_00306 | Type IV secretion system protein VirB6 |
| SOPP22_00307 | Type IV secretion system protein virB8 |
| SOPP22_00308 | Type IV secretion system protein virB9 |
| SOPP22_00309 | Type IV secretion system protein virB10 |
| SOPP22_00310 | Type IV secretion system protein VirB11 |
| SOPP22_00311 | Conjugal transfer protein TraG |
| SOPP22_00312 | DNA primase TraC |
| SOPP22_00313 | hypothetical protein |
| SOPP22_01663 | hypothetical protein |
| SOPP22_01664 | Flagellar assembly protein T, middle domain |
| SOPP22_01665 | hypothetical protein |
| SOPP22_01666 | hypothetical protein |
| SOPP22_01667 | hypothetical protein |
| SOPP22_01668 | Aspartate-semialdehyde dehydrogenase 2 |
| SOPP22_01669 | Erythronate-4-phosphate dehydrogenase |
| SOPP22_01670 | ComE operon protein 1 |
| SOPP22_01671 | ComE operon protein 1 |
| SOPP22_01672 | L-methionine gamma-lyase |
| SOPP22_01673 | hypothetical protein |
| SOPP22_01674 | hypothetical protein |
| SOPP22_01675 | Phage shock protein C |
| SOPP22_01676 | Phage shock protein B |
| SOPP22_02189 | Integrase core domain protein |
| SOPP22_02190 | Putative penicillin-binding protein PbpX |
| SOPP22_02191 | Zeta toxin |
| SOPP22_02192 | hypothetical protein |
| SOPP22_02193 | Tyrosine recombinase XerD |
| SOPP22_02194 | putative nucleotide-binding protein containing TIR-like domain protein |
| SOPP22_02195 | DNA-invertase hin |
| SOPP22_02196 | ParB-like nuclease domain protein |
| SOPP22_02197 | hypothetical protein |
| SOPP22_02198 | hypothetical protein |
| SOPP22_02199 | hypothetical protein |
| SOPP22_02200 | hypothetical protein |
| SOPP22_02202 | 2',3'-cyclic-nucleotide 2'-phosphodiesterase/3'-nucleotidase |
| SOPP22_02203 | hypothetical protein |
| SOPP22_02449 | hypothetical protein |
| SOPP22_02448 | hypothetical protein |
| SOPP22_02447 | hypothetical protein |
| SOPP22_02446 | hypothetical protein |
| SOPP22_02445 | hypothetical protein |
| SOPP22_02444 | DNA-invertase hin |
| SOPP22_02443 | hypothetical protein |
| SOPP22_02442 | hypothetical protein |
| SOPP22_02441 | Putative phage integrase |
| SOPP22_02733 | hypothetical protein |
| SOPP22_02734 | hypothetical protein |
| SOPP22_02735 | Tyrosine recombinase XerC |
| SOPP22_02736 | hypothetical protein |
| SOPP22_02737 | Cbb3-type cytochrome c oxidase subunit CcoN1 |
| SOPP22_02738 | Cytochrome C oxidase, mono-heme subunit/FixO |
| SOPP22_02739 | Cbb3-type cytochrome oxidase component FixQ |
| SOPP22_02740 | Cbb3-type cytochrome c oxidase subunit CcoP2 |
| SOPP22_02741 | FixH |
| SOPP22_02849 | hypothetical protein |
| SOPP22_02850 | Transposase IS116/IS110/IS902 family protein |
| SOPP22_02851 | hypothetical protein |
| SOPP22_02852 | hypothetical protein |
| SOPP22_02853 | hypothetical protein |
| SOPP22_02854 | RES domain protein |
| SOPP22_02855 | hypothetical protein |
| SOPP22_02856 | hypothetical protein |
| SOPP22_02857 | hypothetical protein |
| SOPP22_02858 | hypothetical protein |
| SOPP22_02859 | hypothetical protein |
| SOPP22_02860 | hypothetical protein |
| SOPP22_02861 | hypothetical protein |
| SOPP22_02862 | hypothetical protein |
| SOPP22_02863 | Type II secretion system protein G |
| SOPP22_02864 | hypothetical protein |
| SOPP22_02865 | hypothetical protein |
| SOPP22_02866 | hypothetical protein |
| SOPP22_02867 | hypothetical protein |
| SOPP22_02868 | hypothetical protein |
| SOPP22_02869 | Acetyltransferase (GNAT) domain protein |
| SOPP22_03177 | hypothetical protein |
| SOPP22_03178 | hypothetical protein |
| SOPP22_03179 | Tetratricopeptide repeat protein |
| SOPP22_03180 | hypothetical protein |
| SOPP22_03181 | hypothetical protein |
| SOPP22_03182 | hypothetical protein |
| SOPP22_03183 | hypothetical protein |
| SOPP22_03184 | hypothetical protein |
| SOPP22_03185 | hypothetical protein |
| SOPP22_03186 | hypothetical protein |
| SOPP22_03187 | hypothetical protein |
| SOPP22_03188 | hypothetical protein |
| SOPP22_03189 | Trypsin |
| SOPP22_03190 | hypothetical protein |
| SOPP22_03191 | hypothetical protein |
| SOPP22_03192 | hypothetical protein |
| SOPP22_03193 | hypothetical protein |
| SOPP22_03194 | hypothetical protein |
| SOPP22_03195 | hypothetical protein |
| SOPP22_03196 | Integrase core domain protein |
| SOPP22_03197 | hypothetical protein |
| SOPP22_03294 | Phage integrase family protein |
| SOPP22_03295 | hypothetical protein |
| SOPP22_03296 | hypothetical protein |
| SOPP22_03297 | hypothetical protein |
| SOPP22_03298 | hypothetical protein |
| SOPP22_03299 | hypothetical protein |
| SOPP22_03300 | hypothetical protein |
| SOPP22_03301 | hypothetical protein |
| SOPP22_03302 | hypothetical protein |
| SOPP22_03303 | hypothetical protein |
| SOPP22_03304 | hypothetical protein |
| SOPP22_03305 | AAA domain protein |
| SOPP22_03306 | Lipocalin-like domain protein |
| SOPP22_03307 | hypothetical protein |
| SOPP22_03308 | hypothetical protein |
| SOPP22_03309 | hypothetical protein |
| SOPP22_03310 | group II intron reverse transcriptase/maturase |
| SOPP22_03569 | hypothetical protein |
| SOPP22_03568 | hypothetical protein |
| SOPP22_03567 | hypothetical protein |
| SOPP22_03566 | hypothetical protein |
| SOPP22_03565 | hypothetical protein |
| SOPP22_03564 | hypothetical protein |
| SOPP22_03563 | hypothetical protein |
| SOPP22_03562 | hypothetical protein |
| SOPP22_03561 | hypothetical protein |
| SOPP22_03560 | hypothetical protein |
| SOPP22_03559 | hypothetical protein |
| SOPP22_03558 | hypothetical protein |
| SOPP22_03557 | Cupin domain protein |
| SOPP22_03556 | Proton/glutamate-aspartate symporter |
