# Supplementary material for: Whole-Genome Comparisons Among the Genus Shewanella Reveal the Enrichment of Genes Encoding Ankyrin-Repeats Containing Proteins in Sponge-Associated Bacteria
Source: Front Microbiol. 2019 Feb 6;10:5. doi: 10.3389/fmicb.2019.00005 (PMC6372511; doi:10.3389/fmicb.2019.00005)
Supplement: Supplementary file 8 [file Table_8.DOCX]

**Table S8**. Detected genomic islands in the genome of a sponge-associated *Shewanella* *spongiae* KCTC 22492. Genes encoding the ankyrin repeat-containing proteins are shaded in grey.

| **Locus ID** | **Product** |
| --- | --- |
| SPG_00084 | hypothetical protein |
| SPG_00085 | hypothetical protein |
| SPG_00086 | Acetyltransferase (GNAT) family protein |
| SPG_00087 | Group II intron-encoded protein LtrA |
| SPG_00088 | Pentatricopeptide repeat domain protein |
| SPG_00089 | hypothetical protein |
| SPG_00090 | hypothetical protein |
| SPG_00125 | hypothetical protein |
| SPG_00126 | Rhodopirellula transposase DDE domain protein |
| SPG_00127 | hypothetical protein |
| SPG_00128 | hypothetical protein |
| SPG_00129 | hypothetical protein |
| SPG_00131 | Acetyltransferase (GNAT) family protein |
| SPG_00132 | Bacterial Ig-like domain (group 1) |
| SPG_00133 | Proton/glutamate-aspartate symporter |
| SPG_00134 | Inosine-guanosine kinase |
| SPG_00135 | Adenylate kinase |
| SPG_00136 | Putative thioredoxin 2 |
| SPG_00137 | Chaperone protein HtpG |
| SPG_00138 | hypothetical protein |
| SPG_00139 | hypothetical protein |
| SPG_00741 | Inner membrane protein YbaN |
| SPG_00742 | Pentatricopeptide repeat domain protein |
| SPG_00743 | Pentatricopeptide repeat domain protein |
| SPG_00744 | Glutaminase 2 |
| SPG_00745 | Asparagine synthetase B [glutamine-hydrolyzing] |
| SPG_00746 | hypothetical protein |
| SPG_00747 | Putative DNA-binding domain protein |
| SPG_00867 | Rhodopirellula transposase DDE domain protein |
| SPG_00868 | KAP family P-loop domain protein |
| SPG_00869 | 2OG-Fe(II) oxygenase superfamily protein |
| SPG_00870 | hypothetical protein |
| SPG_00871 | Magnesium transport protein CorA |
| SPG_00872 | hypothetical protein |
| SPG_01678 | hypothetical protein |
| SPG_01679 | hypothetical protein |
| SPG_02461 | hypothetical protein |
| SPG_02462 | IS1 transposase |
| SPG_02463 | hypothetical protein |
| SPG_02464 | Pentatricopeptide repeat domain protein |
| SPG_02465 | hypothetical protein |
| SPG_02466 | Transposase |
| SPG_02467 | hypothetical protein |
| SPG_02468 | Serine/threonine-protein kinase StkP |
| SPG_02469 | Pentatricopeptide repeat domain protein |
| SPG_02470 | hypothetical protein |
| SPG_02485 | MAPEG family protein |
| SPG_02486 | hypothetical protein |
| SPG_02487 | UTP--glucose-1-phosphate uridylyltransferase |
| SPG_02488 | Mannosylglucosyl-3-phosphoglycerate phosphatase |
| SPG_02489 | Extracellular ribonuclease |
| SPG_02490 | hypothetical protein |
| SPG_02491 | hypothetical protein |
| SPG_03019 | mRNA interferase toxin RelE |
| SPG_03020 | Antitoxin YafN |
| SPG_03098 | hypothetical protein |
| SPG_03099 | DNA primase TraC |
| SPG_03100 | hypothetical protein |
| SPG_03101 | DNA primase TraC |
| SPG_03182 | DNA-invertase hin |
| SPG_03183 | hypothetical protein |
| SPG_03184 | Catalase |
| SPG_03185 | Ankyrin repeats (3 copies) |
| SPG_03186 | hypothetical protein |
| SPG_03187 | hypothetical protein |
| SPG_03188 | 2-nitroimidazole transporter |
| SPG_03189 | S-formylglutathione hydrolase YeiG |
| SPG_03190 | S-(hydroxymethyl)glutathione dehydrogenase |
| SPG_03191 | HTH-type transcriptional regulator DmlR |
| SPG_03192 | hypothetical protein |
| SPG_03193 | 4Fe-4S dicluster domain protein |
| SPG_03302 | hypothetical protein |
| SPG_03303 | Group II intron-encoded protein LtrA |
| SPG_03359 | hypothetical protein |
| SPG_03360 | hypothetical protein |
| SPG_03361 | ParE toxin of type II toxin-antitoxin system, parDE |
| SPG_03362 | Tyrosine recombinase XerD |
| SPG_03363 | hypothetical protein |
| SPG_03364 | ATP-dependent RecD-like DNA helicase |
| SPG_03365 | hypothetical protein |
| SPG_03366 | T-DNA border endonuclease VirD2 |
| SPG_03367 | hypothetical protein |
| SPG_03368 | hypothetical protein |
| SPG_03369 | hypothetical protein |
| SPG_03370 | hypothetical protein |
| SPG_03371 | hypothetical protein |
| SPG_03372 | hypothetical protein |
| SPG_03422 | hypothetical protein |
| SPG_03423 | hypothetical protein |
| SPG_03424 | hypothetical protein |
| SPG_03425 | hypothetical protein |
| SPG_03426 | hypothetical protein |
| SPG_03427 | RNA ligase |
| SPG_03428 | hypothetical protein |
| SPG_03429 | Sulfite reductase [NADPH] flavoprotein alpha-component |
| SPG_03452 | RDD family protein |
| SPG_03501 | Nuclease-related domain protein |
| SPG_03502 | Spermine/spermidine acetyltransferase |
| SPG_03503 | hypothetical protein |
| SPG_03505 | Zeta toxin |
| SPG_03506 | hypothetical protein |
| SPG_03507 | Putative DNA repair helicase RadD |
| SPG_03508 | Peptide chain release factor 2 |
| SPG_03509 | RNA-splicing ligase RtcB |
| SPG_03510 | Tyrosine recombinase XerD |
| SPG_03519 | Pentapeptide repeats (9 copies) |
| SPG_03520 | Ribosomal RNA large subunit methyltransferase J |
| SPG_03521 | hypothetical protein |
| SPG_03522 | hypothetical protein |
| SPG_03523 | Peptidase family M1 domain protein |
| SPG_03524 | hypothetical protein |
| SPG_03555 | hypothetical protein |
| SPG_03556 | hypothetical protein |
| SPG_03557 | Protein kinase domain protein |
| SPG_03558 | hypothetical protein |
| SPG_03559 | hypothetical protein |
| SPG_03560 | hypothetical protein |
| SPG_03561 | Pentatricopeptide repeat domain protein |
| SPG_03579 | hypothetical protein |
| SPG_03580 | Transposase DDE domain protein |
| SPG_03588 | hypothetical protein |
| SPG_03589 | Transcriptional regulator PadR-like family protein |
| SPG_03590 | hypothetical protein |
| SPG_03591 | Bis(5'-nucleosyl)-tetraphosphatase, symmetrical |
| SPG_03593 | Pentatricopeptide repeat domain protein |
| SPG_03710 | IS1 transposase |
| SPG_03711 | hypothetical protein |
| SPG_03731 | Ankyrin repeats (3 copies) |
| SPG_03732 | Ankyrin repeats (3 copies) |
| SPG_03733 | hypothetical protein |
| SPG_03754 | Pentatricopeptide repeat domain protein |
| SPG_03755 | Peptidase family S41 |
| SPG_03765 | hypothetical protein |
| SPG_03766 | hypothetical protein |
| SPG_03767 | Ribosomal RNA large subunit methyltransferase F |
| SPG_03768 | hypothetical protein |
| SPG_03769 | hypothetical protein |
| SPG_03778 | hypothetical protein |
| SPG_03779 | N-acetylmuramic acid 6-phosphate etherase |
| SPG_03780 | hypothetical protein |
| SPG_03781 | hypothetical protein |
| SPG_03782 | Ankyrin repeats (3 copies) |
| SPG_03785 | hypothetical protein |
| SPG_03786 | Bacterial antitoxin of ParD toxin-antitoxin type II system and RHH |
| SPG_03787 | Toxin ParE1 |
| SPG_03788 | Group II intron-encoded protein LtrA |
| SPG_03797 | hypothetical protein |
| SPG_03798 | hypothetical protein |
| SPG_03799 | hypothetical protein |
| SPG_03805 | hypothetical protein |
| SPG_03806 | Ankyrin repeats (3 copies) |
| SPG_03807 | hypothetical protein |
| SPG_03808 | Pentatricopeptide repeat domain protein |
| SPG_03817 | hypothetical protein |
| SPG_03818 | hypothetical protein |
| SPG_03819 | hypothetical protein |
| SPG_03820 | hypothetical protein |
| SPG_03824 | Pentatricopeptide repeat domain protein |
| SPG_03842 | hypothetical protein |
| SPG_03843 | Antitoxin Phd_YefM, type II toxin-antitoxin system |
| SPG_03844 | Toxin RelE2 |
| SPG_03845 | DDE superfamily endonuclease |
| SPG_03854 | IS1 transposase |
| SPG_03855 | UDP-N-acetylglucosamine 4-epimerase |
| SPG_03859 | hypothetical protein |
| SPG_03860 | hypothetical protein |
| SPG_03861 | Ankyrin repeats (3 copies) |
| SPG_03866 | hypothetical protein |
| SPG_03867 | Pentatricopeptide repeat domain protein |
| SPG_03869 | hypothetical protein |
| SPG_03870 | hypothetical protein |
| SPG_03871 | hypothetical protein |
| SPG_03872 | hypothetical protein |
| SPG_03873 | sulfur transport |
| SPG_03874 | hypothetical protein |
| SPG_03881 | hypothetical protein |
| SPG_03883 | hypothetical protein |
| SPG_03884 | hypothetical protein |
| SPG_03888 | hypothetical protein |
| SPG_03889 | hypothetical protein |
| SPG_03890 | PPR repeat family protein |
| SPG_03891 | hypothetical protein |
| SPG_03892 | hypothetical protein |
| SPG_03906 | T-DNA border endonuclease VirD2 |
| SPG_03907 | Bacterial mobilization protein (MobC) |
| SPG_03908 | Antitoxin HigA |
| SPG_03909 | mRNA interferase toxin HigB |
| SPG_03910 | hypothetical protein |
| SPG_03911 | hypothetical protein |
| SPG_03913 | hypothetical protein |
| SPG_03915 | Ankyrin repeats (3 copies) |
| SPG_03916 | hypothetical protein |
| SPG_03917 | hypothetical protein |
| SPG_03918 | hypothetical protein |
| SPG_03921 | hypothetical protein |
| SPG_03922 | putative type I restriction enzymeP M protein |
| SPG_03923 | hypothetical protein |
| SPG_03924 | Helix-turn-helix domain protein |
| SPG_03933 | hypothetical protein |
| SPG_03935 | hypothetical protein |
| SPG_03936 | Transposase IS66 family protein |
| SPG_03937 | hypothetical protein |
| SPG_03938 | hypothetical protein |
| SPG_03939 | HTH-type transcriptional regulator ImmR |
| SPG_03940 | hypothetical protein |
| SPG_03941 | hypothetical protein |
| SPG_03942 | hypothetical protein |
| SPG_03943 | hypothetical protein |
| SPG_03944 | hypothetical protein |
| SPG_03945 | hypothetical protein |
| SPG_03946 | hypothetical protein |
| SPG_03961 | hypothetical protein |
| SPG_03962 | hypothetical protein |
| SPG_03963 | hypothetical protein |
| SPG_03978 | hypothetical protein |
| SPG_03979 | hypothetical protein |
| SPG_03980 | Transposase IS66 family protein |
| SPG_03988 | hypothetical protein |
| SPG_03993 | DDE_Tnp_1-associated |
| SPG_03996 | Lipoprotein NlpI |
| SPG_04007 | Transposase IS116/IS110/IS902 family protein |
| SPG_04010 | Rhodopirellula transposase DDE domain protein |
| SPG_04011 | hypothetical protein |
| SPG_04012 | Winged helix-turn helix |
| SPG_04016 | Protein kinase domain protein |
