# Supplementary material for: Whole-Genome Comparisons Among the Genus Shewanella Reveal the Enrichment of Genes Encoding Ankyrin-Repeats Containing Proteins in Sponge-Associated Bacteria
Source: Front Microbiol. 2019 Feb 6;10:5. doi: 10.3389/fmicb.2019.00005 (PMC6372511; doi:10.3389/fmicb.2019.00005)
Supplement: Supplementary file 9 [file Data_Sheet_1.PDF]

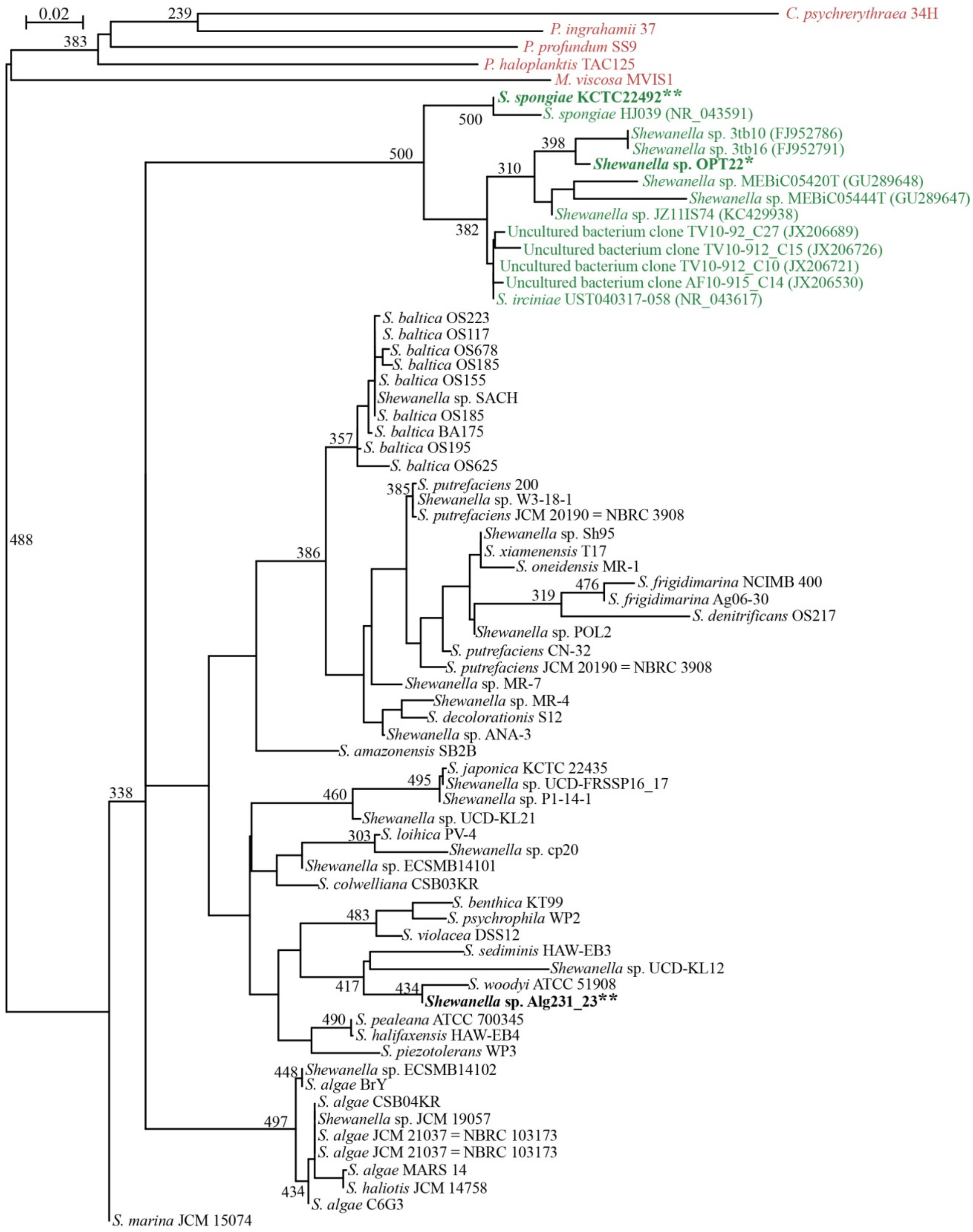

Figure S1. Maximum-likelihood phylogenetic tree of the genus Shewanella using the 16S rRNA gene sequences. Strains in bold denote the only available genomes of sponge-associated *Shewanella* that are used in this study. The *Shewanella* sp. OPT22 sequenced in this study is highlighted by a single asterisk, grouped with other sponge-associated *Shewanella* strains (sponge-specific cluster in green). The strains marked with two asterisks denote another sponge-associated *Shewanella*. Bootstrap support values greater than 200 (500 BS replicates) are shown at each node. The species highlighted in red were used as outgroups.
