# Supplementary material for: Whole-Genome Comparisons Among the Genus Shewanella Reveal the Enrichment of Genes Encoding Ankyrin-Repeats Containing Proteins in Sponge-Associated Bacteria
Source: Front Microbiol. 2019 Feb 6;10:5. doi: 10.3389/fmicb.2019.00005 (PMC6372511; doi:10.3389/fmicb.2019.00005)
Supplement: Supplementary file 10 [file Data_Sheet_2.PDF]

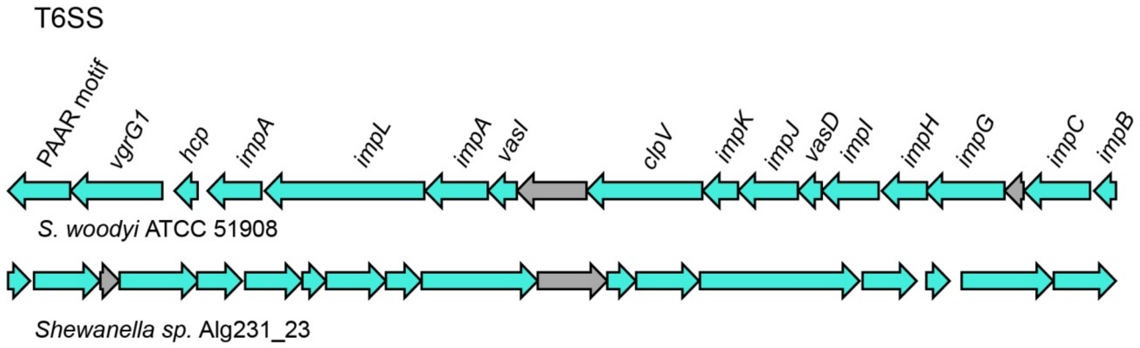

Figure S2. Syntenic organization of type VI secretion system detected in two closely related strains, *S. woodyi* ATCC 51908 and *Shewanella* sp. Alg231_23 isolated from a squid and a sponge respectively. Grey color denotes the unrelated genes detected within the T6SS gene cluster.
