# Supplementary material for: Whole-Genome Comparisons Among the Genus Shewanella Reveal the Enrichment of Genes Encoding Ankyrin-Repeats Containing Proteins in Sponge-Associated Bacteria
Source: Front Microbiol. 2019 Feb 6;10:5. doi: 10.3389/fmicb.2019.00005 (PMC6372511; doi:10.3389/fmicb.2019.00005)
Supplement: Supplementary file 11 [file Data_Sheet_3.PDF]

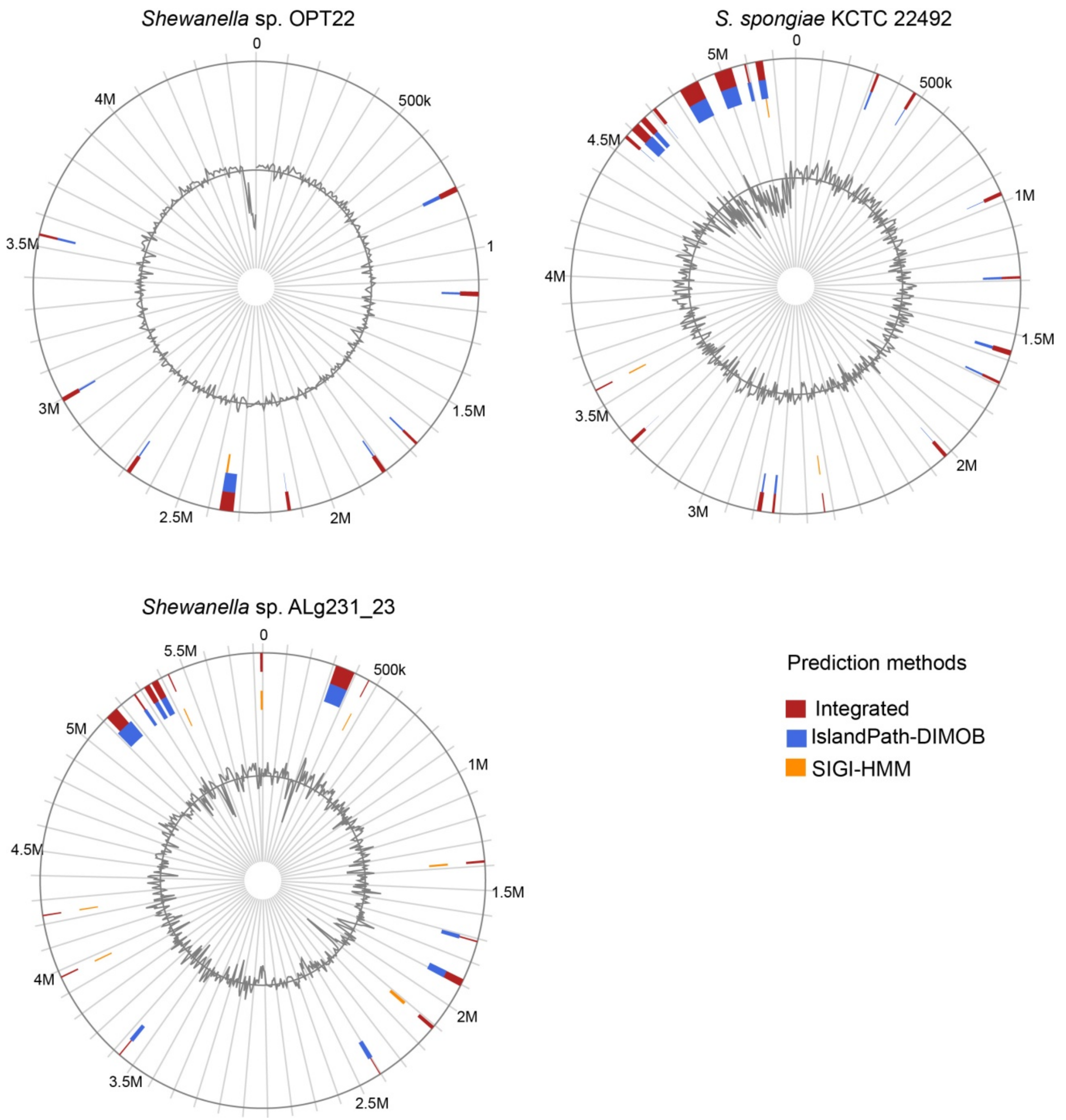

Figure S3. Predicted genomic islands (GIs) in the genomes of the three sponge-associated *Shewanella* species. Color codes inside the ring structure represent the GIs detected by each prediction methods.
